# Exploring the role of ecology and social organisation in agropastoral societies: A Bayesian network approach

**DOI:** 10.1371/journal.pone.0276088

**Published:** 2022-10-26

**Authors:** Olga Palacios, Juan Antonio Barceló, Rosario Delgado

**Affiliations:** 1 Department of Prehistory, Laboratory of Quantitative Archaeology, Universitat Autònoma de Barcelona, Cerdanyola del Vallès, Barcelona, Spain; 2 Department of Mathematics, Universitat Autònoma de Barcelona, Cerdanyola del Vallès, Barcelona, Spain; Universidade Federal de Pernambuco, BRAZIL

## Abstract

The present contribution focuses on investigating the interaction of people and environment in small-scale farming societies. Our study is centred on the particular way settlement location constraints economic strategy when technology is limited, and social division of work is not fully developed. Our intention is to investigate prehistoric socioeconomic organisation when farming began in the Old World along the Levant shores of Iberian Peninsula, the Neolithic phenomenon. We approach this subject extracting relevant information from a big set of ethnographic and ethnoarchaeological cases using Machine Learning methods. This paper explores the use of Bayesian networks as explanatory models of the independent variables–the environment- and dependent variables–social decisions-, and also as predictive models. The study highlights how subsistence strategies are modified by ecological and topographical variables of the settlement location and their relationship with social organisation. It also establishes the role of Bayesian networks as a suitable supervised Machine Learning methodology for investigating socio-ecological systems, introducing their use to build useful data-driven models to address relevant archaeological and anthropological questions.

## Introduction

A socio-ecological system can be described as a structure defined by the interaction among social behaviours (e.g., subsistence strategies and social organisation) and ecological features of the location where social action took place (temperature, pluviometry, topography, soil features, etc.) [1]. This approach has been implemented in Archaeology and Anthropology among other Social Sciences to explore questions concerning how modern and ancient societies–even prehistoric- lived in the past and in the present and managed their environmental resources. Socio-Ecological System theory can be considered as a response to the limitations of traditional research approaches that addressed social modelling from reductionist assumptions [2]. From this perspective, past communities are not investigated in isolation but considering the environmental and ecological characteristics that surrounded them and ultimately resulted from their interaction with the natural setting. Thus, the role of human agency for modifying and transforming the environment is recognised like the importance of the landscape to define human activities.

The debate concerning the relationship and interaction of early agropastoral communities with the environment has a long trajectory in archaeology (some examples include [3–6]). To address this topic, Niche Construction Theory has gained relevance in the last decades to study the plant and animal domestication process [7–11]. In this line, Smith argued in 2011 that early Neolithic communities were small-scale farming communities shared a similar behaviour: most of them had well-defined resource catchment/s area/s; they knew their ecosystem well and they constantly adapted to their own caused environmental modification [7]. It would have resulted in an increase in the probability of survival at those. However, probably not all the intervening factors (i.e., resource availability, prior knowledge, size of catchment area) had the same impact when early farming communities decided of what foodstuff was the best for their current situation. In fact, it was probably different for different communities, as additional particular variables may have been relevant given local conditions and circumstances.

We assume past communities constructed their niche by taking social decisions about how their environmental conditions could be modified to increase the chances of survival. There are numerous examples of prehistoric activities modifying the landscape with water [12] or fire to practice slash-and-burn agricultural method [13, 14] or vegetation clearance for procuring pastures nearby [15]. These behaviours and cultural processes [16], not only modified the genetics of the species found in the niches (a clear example of that is genetic change experimented by animals and plants during domestication), but also the way people lived, their households, their types of settlements, their relationship, etc. It was a reciprocal transformational process.

In this paper, we are interested in studying how prehistoric small-scale food producers [7] took social and economic decisions–where to settle- from their own observation of climatic and ecological features around them, the influence of the environment on their survival expectations and their knowledge of the possible consequences of their activity on that environment. We focus our study on the maintenance of agropastoralism lifestyle of small-scale farming communities rather than investigating the origins of agriculture in itself. Our research should be considered as a new argument within the current trend of studies towards how early agropastoral economies were configured [17–19]. Beyond exploring a particular historical case, Old World Neolithic, for instance, we are interested in global dynamics, that could be of interest to understand different settlement patterns in different parts of the world in different chronologies. We would like to identify if there was some form of regularity or communality in potential socioeconomic behaviours of small-scale agropastoral communities that could be more likely to be present in some landscapes rather than in others. The goal is then to contribute to the understanding of eco-evolutionary relationship between the environment and people in the Past and in the Present, when industrialisation and market relationships are absent. To achieve this objective, we are asking two fundamental research questions:

**Q1)** Do ecological features of settlement location and/or social organisation constrained the type and intensity of subsistence strategies?
**Q2)** Do ecological features of settlement location and/or the type and intensity of subsistence strategies constrained social organisation?

Our research analyses the importance of the landscape to understand economic dynamics in communities with simple social organisation and low efficient technologies, and the effects of social organisation in understanding the transformation experimented by landscape. We understand that the impact of society on the landscape and the landscape on society imply the study of multiple statistically causal (direct) and non-causal (indirect) links between independent variables–landscape- and dependent variables–the human group- [20, 21]. These relationships have been analysed using the standard way of describing social decisions and natural environmental settings in small-scale farming communities in ethnology, ethnohistory and archaeology (S1 Table). Among the landscape factors retained for analysis, we can mention elevation, slope, temperature variation, precipitation variation, natural soil productivity depending on soil composition, etc. Social decisions can be grouped into three main topics: 1) the strategy adopted to acquire subsistence (agriculture, animal husbandry, hunting, gathering, fishing); 2) features of the social organisation (community size, kind of settlement, local group organisation, household organisation) and 3) social decisions that can be adopted when survival is at risk (for example, in times of food scarcity) (Table 1). For instance, sometimes a human community can decide an economic strategy towards crop specialisation to compensate for diminishing marginal returns [22, 23], or, alternatively, it can decide a diversification strategy for the same reason [24, 25]. Exchange in goods and/or food can increase subsistence acquisition [26]; people displacement–migration- can be decided to better share existing resources [22, 27, 28], etc.

**Table 1:** Summary of relevant variables to consider for modelling socio-ecological systems.

INDEPENDENT VARIABLES	DEPENDENT VARIABLES		
Ecology	Subsistence strategies	Social Organisation	Social decisions
Landscape	Agriculture	Community size	None
Distance to coast	Animal husbandry	Settlement types	Resource diversification
Elevation	Hunting	Community organisation	Crop specialisation
Slope	Gathering	Household organisation	Foraging intensification
Annual mean temperature	Fishing		Storage
CV Annual temperature			Transhumance
Monthly mean Precipitation			Temporal / Permanent migration
CV Annual precipitation			Exchange in-/out-settlement
Monthly primary net soil productivity			Reciprocity
CV Primary net soil productivity			

We have considered the necessity to include in our dataset the social decision of not taking any action in front of scarcity (‘None’ in our dataset). This kind of behaviour has been described by prior authors as ‘supply-induced scarcity’ [25, 27, 29] in which communities reduce their food intake and suffer some periods of hunger. These kind of behaviour has been identified in some agropastoral communities such as the Anaguta [S1 File: References 98, 99], the Chucki [S1 File: Reference 150], the Lovedu / Balobedu [S1 File: References 267, 268], the Maasai [S1 File: References 273, 277] and the Mambila / Mambilla [S1 File: References 281, 282]. For example, Spencer (1988) described this social decision in the Maasai of Matapata as:

“*The problem of drought is never quite resolved*, *but as Matapata view their mode of adaptation to their ecological nicge*, *the benefits for those who survive and thrive are prefereable to any alternative*”. [S1 File: Reference 273]

In this paper, we show how we can define hypothetically probabilistic relationships between landscape factors and different social decisions. For instance, we analyse how the high elevation of a settlement area may have constrained the adoption of resource diversification or crop specialisation; whether the role of annual precipitation of the region to be settled has had any effect on the size for the human group that finally settled there.

The amount of influence and effect a variable has on any other can be expressed in probabilistic terms. We are looking for regularities expressed probabilistically to be able to predict and explain ethnological/archaeological observations. For instance, imagine we have documented a Neolithic settlement not far from a source of water, on the plain, in a region of low temperature annual variation (estimated from a paleo temperature record), and where grassland was the dominant vegetation. Built on that observation, we would like to predict whether this community practised at that time an agriculture based on resource diversification without crop specialisation and a high level of external exchange. To formulate those predictions, we need to know the probability with which values of different variables may appear together. A usual source of error in this kind of studies lies on the assumption that input variables–climatic and ecological features of settlement location- are independent among them, and that all of them have a similar impact determining the output–the social decision. On the contrary, features like water, insolation, temperature, natural soil productivity, etc. are interrelated in a complex and non-linear way with feedback across variables defining the social behaviour [30].

The necessary probabilistic thresholds can be defined in terms of inductive regularities extracted from an exhaustive data set of well-known and described cross-cultural case studies [31–36], provided the database is big enough and it does not alter the expected variability of human decisions. The application of trans-historic and cross-cultural data is particularly employed to evaluate hypotheses about Prehistory since the validation of social hypotheses about the past is often challenging [37, 38]. Generalising from a rationally built set of particular cases is the most usual way to interpret human behaviour [39, 40]. By learning what is common in living societies, we can mitigate the lack of this type of knowledge in the archaeological record.

The validity of ethnographic analogy has been strongly debated [41–43] because it employs the information of modern societies to interpret a possibly imagined past. Despite its inherent subjectivity, this kind of inverse reasoning approach can aid our interpretation of the archaeological record by providing information about what sort of behaviours could have been practised in the past. To measure the most probable behaviours, we need to collect the higher number of cases as possible to extract meaningful regularities to consider all the potential underlying variations of social decisions. This issue has been identified by many authors, and it constitutes the basis for modern ethnoarchaeological studies [21, 44–46] which, again, does not attempt to draw direct analogies from the present to the past, but explore possible behaviours that may have been practiced in the past. In our case, to explore the probable communalities in small-scale communities, similar to those that may have existed in Prehistory, we have limited the learning data set to farming communities settled in not heavily transformed landscapes, practicing a mixed farming economy with low-efficiency technology and small quantities of human work [17, 47–49]. This is the classical assumption of Prehistoric Neolithic Economies [3, 50–52].

Among the many possible statistical and computational methods to compute similarity relationships and communalities among particular ethnographic cases, we have decided to use Machine Learning methods since they allow building models based on empirical data without prior assumptions. The resulting model is objective and captures the relationships between the variables in the collected data, without external intervention. Since the model is built from the dataset autonomously, it will be automatically relearned from successive data updates (whence the terminology “machine learning”). In this, it differs from a classical statistical model, which only captures the information of the moment and if new data is added to the dataset, the model is not automatically updated accordingly, but rather must be redesigned from scratch.

Many different Machine Learning methods have been employed to build socio-ecological systems, centred on understanding how people managed their environment [18, 19, 34, 53]. Notwithstanding, the number of archaeological studies that use the Machine Learning methodology is still a minority compared to those that use other quantitative and/or qualitative methods. An additional problem is that many times the resulting model is just a “black box”, suitable for some predictive tasks, but without explanatory capabilities, since the way the input is related to the output is not visible to the user.

To alleviate this deficiency, in this paper, we propose the use of *Bayesian networks* (BNs), which are a kind of supervised algorithm [54]. This method has been previously applied in other research studies for designing conceptual models [55, 56] but not as the machine learning method that it really is (at least that the authors are aware of). Other studies that have explored past socio-ecological systems from the machine learning approach have employed other algorithms, such as logistic regression [57], deep learning [58], support vector machine [19], random forest [59] or combined some of these algorithms [18, 60].

The remainder of the paper is structured as follows: the Materials and Methods section deals with the data and the method employed in the study, specifying the data collection process and the model building and implementation. In the Results section, the results obtained are presented and discussed in the Discussion section. Finally, the article summarises the most relevant insights of the study in the Conclusions section.

## Materials and methods

### Data collection, cleaning, and pre-processing

To predict among the different possible ways small-scale human groups may have decided where to settle, we have investigated a trans-historical and cross-cultural dataset including 173 case studies collected from two open access repositories: D-PLACE [61] and The Human Relations Area Files (eHRAF) [62] (S1 Dataset). We have selected in both repositories cases for which detailed information for our list of variables existed and could be checked in the literature. Most data come from D-PLACE in first instance, and the Human Relations Area Files were consulted to check the information by reviewing the monographs of each community (S2 Dataset). Cases were deleted in case of inconsistency between these two repositories. Ethnographical cases were selected according to two criteria: *small-scale* and *farming* societies. That means, human groups–settlements- of less than 1000 inhabitants, and societies acquiring more than 50% of their subsistence from farming strategy: agricultural, and animal husbandry, with other additional resources from fishing, foraging, and hunting. In so doing, we have tried to minimise analogical bias by focusing our research on the most similar cases to the assumed target: Early Neolithic small settlements, where farming has been empirically established–domesticated plants and animals-, although there is additional archaeological evidence of alternative economic strategies. The resulting data set may be considered relatively small. It is however very coherent, and the underlying variation is meaningful and clearly related with the different ways these kind of societies exploited their hinterland. We have privileged the quality and reliability of the sample rather than the number and exhaustivity, provided social variation is not affected by the selection process.

Values for a total of 30 variables have been carefully recorded for each ethnographic case, based on the preliminary selection of independent and dependent variables (summarised in Table 2, see S2 Table for more detailed information). The quality of the detailed information in original sources is inconstant, and therefore we have standardised descriptions. Because usual Bayesian Networks link categorical variables, we have discretised quantitative values into uniform bins.

**Table 2:** Qualitative variables and their categorical values investigated in this research. It contains the 30 variables and their values. Ecological characteristics of settlement location and its catchment area are defined in nominal scales using integrative categories.

Information	Variable	Values after discretisation	Variability range before discretization
**Environmental characteristics**	Landscape	*Forest*	Tropical & Subtropical Dry Broadleaf Forests; Tropical & Subtropical Moist Broadleaf Forests; Tropical & Subtropical Coniferous Forests; Temperate Broadleaf & Mixed Forests; Temperate Conifer Forests; Boreal Forests/Taiga; Mediterranean Forests, Woodlands & Scrub
		*Grassland*	Tropical & Subtropical Grasslands; Temperate Grasslands; Flooded Grasslands & Savannas; Montane Grasslands & Shrublands
		*Aquatic*	Ice; Inland water
		*Tundra*	
		*Desert*	Savannas & Shrublands; Deserts & Xeric Shrublands
	Distance to coast (km)	*short distance*	< 10
		*medium distance*	10–50
		*long distance*	>50
	Elevation (m)	*low*	< 300
		*medium*	300–1000
		*high*	>1000
	Slope (°)	*low*	< 0.75
		*medium*	0.75–2.5
		*high*	>2.5
	Annual mean temperature (°C/month)	*low*	< 5
		*medium*	5–20
		*high*	> 20
	Coefficient of variation temperature (°C/month)	*low*	< 0.05
		*medium*	0.05–0.15
		*high*	> 0.15
	Monthly mean precipitation (ml/m2/month)	*low*	<95000
		*medium*	95000–130000
		*high*	>130000
	Coefficient of variation precipitation (ml/m2/month)	*low*	<0.06
		*medium*	0.06–0.08
		*high*	>0.08
	Monthly mean net primary production (gC/m2/month)	*low*	< 1
		*medium*	1–3
		*high*	>3
	Coefficient of variation net primary production (gC/m2/month)	*low*	<0.03
		*medium*	0.03–0.05
		*high*	>0.05
**Subsistence strategies**	Hunting (%)	*None*	0–5
		*<25*	6–15; 16–25
		*> = 25*	26–35; 36–45; 46–55; 56–65; 66–75; 76–85; 86–100
	Gathering (%)	*None*	0–5
		*<25*	6–15; 16–25
		*> = 25*	26–35; 36–45; 46–55; 56–65; 66–75; 76–85; 86–100
	Animal husbandry (%)	*None*	0–5
		*<25*	6–15; 16–25
		*> = 25*	26–35; 36–45; 46–55; 56–65; 66–75; 76–85; 86–100
	Fishing (%)	*None*	0–5
		*<25*	6–15; 16–25
		*> = 25*	26–35; 36–45; 46–55; 56–65; 66–75; 76–85; 86–100
	Agriculture (%)	*None*	0–5
		*<55*	6–15; 16–25; 26–35; 36–45; 46–55
		*>55*	56–65; 66–75; 76–85; 86–100
**Social organisation**	Community size	*<200*	<50; 50–99; 100–199
		*> = 200*	200–399; >400
	Settlement types	*Camp*	
		*Homesteads*	
		*Hamlet*	
		*Village*	
	Community organisation	*NA*	
		*Clan communities*	
		*No exogamous clans*	
	Household organisation	*Small extended*	
		*Large Extended*	
		*Nuclear*	
**Social decisions**	None	*Yes/No*	
	Resource diversification	*Yes/No*	
	Crop specialisation	*Yes/No*	
	Foraging resources intensification	*Yes/No*	
	Storage	*Yes/No*	
	Transhumance	*Yes/No*	
	Temporal migration	*Yes/No*	
	Permanent migration	*Yes/No*	
	Exchange out-settlement	*Yes/No*	
	Exchange in-settlement	*Yes/No*	
** **	Reciprocity	*Yes/No*	

In Machine Learning, training sets are usually larger, often in the category of Big Data. Nothing similar exists in the social domain, where the number of individual cases to be considered for induction and generalisation is by definition reduced. The advantage is coherence of the data set and the possibilities of reducing extrinsic variation. It implies, however, the need of grouping attributes to avoid the risk of over-particularisation.

The way we have integrated some classical environmental characteristics into global categories may seem unclassical, different from what has been applied in other studies. For instance, the category “Forest” in the qualitative variable “Landscape” integrates in the same category environmental settings such as tropical and subtropical dry broadleaf forests, boreal and taiga forest. Nevertheless, differentiating among types of “Forests” is still possible in our model given other variables in the dataset refer to climatic aspects like temperature and precipitation, both intensity and annual variation. Obviously, when grouping apparently different values into global categories we may lose information that could be relevant for characterising the individual characteristics of local ecological niches. It should be taken into account that we are interested in maximising global processes well beyond the local specificities. Given that we have restricted the number of cases for the reinforcement of data reliability, we have been also obliged to reduce the impact of individual details, that make reference to very local aspects. In so doing, we allow the calculation of potential *accurate* predictions, although we lose something in their *precision*. That is, we increase the possibility of finding global processes that may have acted in different contexts and historical scenarios, although such global processes may have had some local differences. Both accuracy and precision reflect how close a prediction is to an actual observation, but accuracy reflects how close a predicted value is to a known or observed value, while precision reflects how reproducible predictions are, even if they are far from the observed value at some particular circumstance.

This approach is necessary for any type of generalisation model. It applies in particular to Bayesian networks, for whose construction we have to estimate from the dataset the probability distribution of each variable conditioned to the possible values of its parents. Therefore, the more different categories the variables have, the more parameters we will have to estimate, for which we would need a dataset with many more cases than the one that we currently have.

Grouping categories and discretizing variables that were quantitative in origin has been carried out using the R software [63]: the function **discretize** of the *arules* R package [64]. Missing values have handled by deleting those variables in which they were very abundant, and some redundant variables were also eliminated. For this, the function **vis_miss** from the *visdat* R package [65] and **gg_miss_var** from the *naniar* R package [66] have been used.

### Bayesian networks

Bayesian Networks are probabilistic graphical models representing the relationships among variables affecting a phenomenon, which can be used for probabilistic inference. For a set of random variables *V* = {X_1_,…,X_n_}, that we assume to be discrete or categorical, a standard BN is a model that represents their joint probability distribution *P*, whose graphical part is a *directed acyclic graph* **G**. The nodes of **G** represent the random variables and the directed arcs among the nodes represent the conditional dependencies (not necessarily causal), which are governed by the *Markov condition*, explained below.

It is said that node *A* is a *parent* of node *B* (and reciprocally, that *B* is a *child* of *A*) if there is a directed arc in **G** from *A* to *B*. We denote by *PA(B)* the set of parents of *B* (it is the empty set if *B* has no parents, and we say that it is a “root” node). If there is a “path” from node *A* to node *B*, that is, a concatenation of directed arcs connecting them, we say that *B* is a *descendant* of *A*. ***Markov condition*** can be expressed as follows: “each variable in *V* is conditionally independent of any of its non-descendants conditioning to the state of all its parents”. Moreover, *P* can be expressed as the product of the conditional distributions of all nodes given the values of their parents, whenever these conditional distributions exist. This is what is known as **chain rule**, formally expressed as follows:

$$P\left(X_{1}=x_{1},\ldots ,X_{n}=x_{n}\right)=\overset{n}{\underset{i=1}{\prod }}P\left(X_{i}=x_{i}/PA(X_{i})\right)$$

for all the possible values of the variables X_1_,…,X_n_ [67]. The chain rule allows to obtain the joint distribution of the variables from the conditional probability table (CPT) of each node conditioned to its parents in **G**, and from the marginal distribution of the root nodes. The probability values of these conditional and marginal distributions are the parameters of the BN to be learned from data, jointly with the structure **G**.

We adopt the *hill climbing greedy search-and-score* structure learning algorithm to learn **G** [54, 67]. This algorithm explores the space of the directed acyclic graphs by single-arc addition, removal, and reversals, to find the structure that maximizes the score function. We will consider two different score functions: *Bayesian Information Criterion* (BIC) [68], and *Akaike Information Criterion* (AIC) [69], both based on the logarithm of the likelihood function but with a term that penalizes for complexity. Since AIC penalizes less, using this score leads to learned Bayesian networks with more connected structure **G**. The parameters are estimated by using the *Maximum Likelihood Estimation* (MLE) method, as usual in statistics.

**Fig 1:**
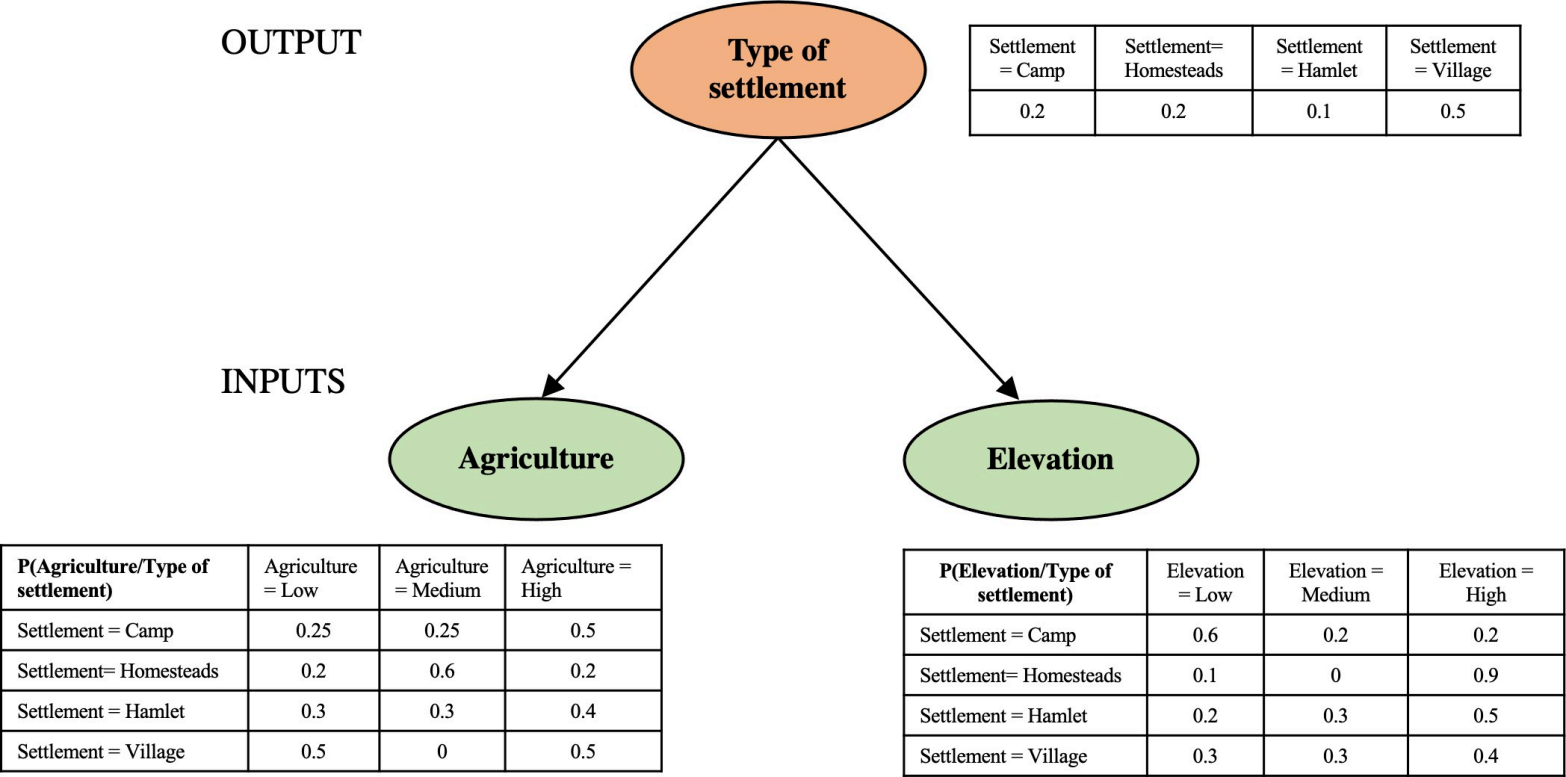
Example of a BN to predict the type of settlement. Type of settlement (output, orange), agriculture and elevation (inputs, green).

Once the predictive model is learned from the data, it can be used to make inferences. Given the evidence corresponding to the values of some of the variables (*input*), a value can be predicted for another of the variables we are interested in (*output*), which will be the most probable value conditioning to the evidence, following the *Maximum A Posteriori* (MAP) criterion. Let us show it with an example of the BN of Fig 1, where the input variables are **Agriculture** an **Elevation** and the output (or class) variable is **Type of settlement**, and each of them have different values: Agriculture has three values (Low, Medium, High); Elevation also has three values (Low, Medium, High); and Type of settlement has four values (Camp, Homesteads, Hamlet, Village). For the input variables we have the CPTs (conditioning to their parent in **G**, which is **Type of settlement**), and for **Type of settlement**, which is a “root” node, we have the table of the marginal distribution.

If the evidence is that **Agriculture** = High and **Elevation** = Low, which is the prediction given by the model (BN) for the class variable **Type of settlement**? We must compute

$$P(Settlement=Village/Agriculture=High,Elevation=Low)=\frac{P(Settlement=Village,Agriculture=High,Elevation=Low)}{P(Agriculture=High,Elevation=Low)}$$ (1)

For the numerator, by using the chain rule:

P(Settlement=Village,Agriculture=High,Elevation=Low)=P(Agriculture=High/Settlement=Village)P(Elevation=Low/Settlement=Village)P(Settlement=Village)=0.5×0.3×0.5=0.075

And in the denominator, we also use the chain rule with the four summands (one for each value of Type of settlement):

P(Agriculture=High,Elevation=Low)=P(Agriculture=High,Elevation=Low,Settlement=Village)+P(Agriculture=High,Elevation=Low,Settlement=Hamlet)+P(Agriculture=High,Elevation=Low,Settlement=Homesteads)+P(Agriculture=High,Elevation=Low,Settlement=Camp)=0.5×0.3×0.5+0.4×0.2×0.1+0.2×0.1×0.2+0.5×0.6×0.2=0.075+0.008+0.004+0.06=0.147

Then, by replacing in (1) we obtain the probability of Type of settlement = Village conditioned to the evidence that Agriculture = High and Elevation = Low

$$P(Settlement=Village/Agriculture=High,Elevation=Low)=\frac{0.075}{0.147}≅0.5102$$

And analogously with the other values of Type of settlement,

$$P(Settlement=Hamlet/Agriculture=High,Elevation=Low)=\frac{0.008}{0.147}≅0.0544$$

$$P(Settlement=Homesteads/Agriculture=High,Elevation=Low)=\frac{0.004}{0.147}≅0.0272$$

$$P(Settlement=Camp/Agriculture=High,Elevation=Low)=\frac{0.06}{0.147}≅0.4082$$

Since the probability of **Type of settlement** = Village conditioning to the evidence is the maximum of the four probabilities, by the MAP criterium the prediction for **Type of settlement** provided by the BN, given the evidence, is Village, with a *confidence level* of 0.5102.

We have selected the method of Bayesian networks for this study because of its advantages over other machine learning methods:

- BNs are “white boxes”, that is, they are interpretable models that can be explained in understandable terms and that transparently describe the relationships and patterns between the variables involved, clearly show how predictions are obtained and what are the influential variables, and help generate insights and perspectives [70–72].
- Their character of graphic models that is given by the *directed acyclic graph*, together with the *Markov condition* and the *Chain rule*, which allows to obtain the joint probability distribution of the variables of the model (and, therefore, any other probability) from the conditional probabilities of each node to its parents [73], make these probabilistic models a versatile, useful, and unique methodology in the current landscape of ML models.

**Fig 2:**
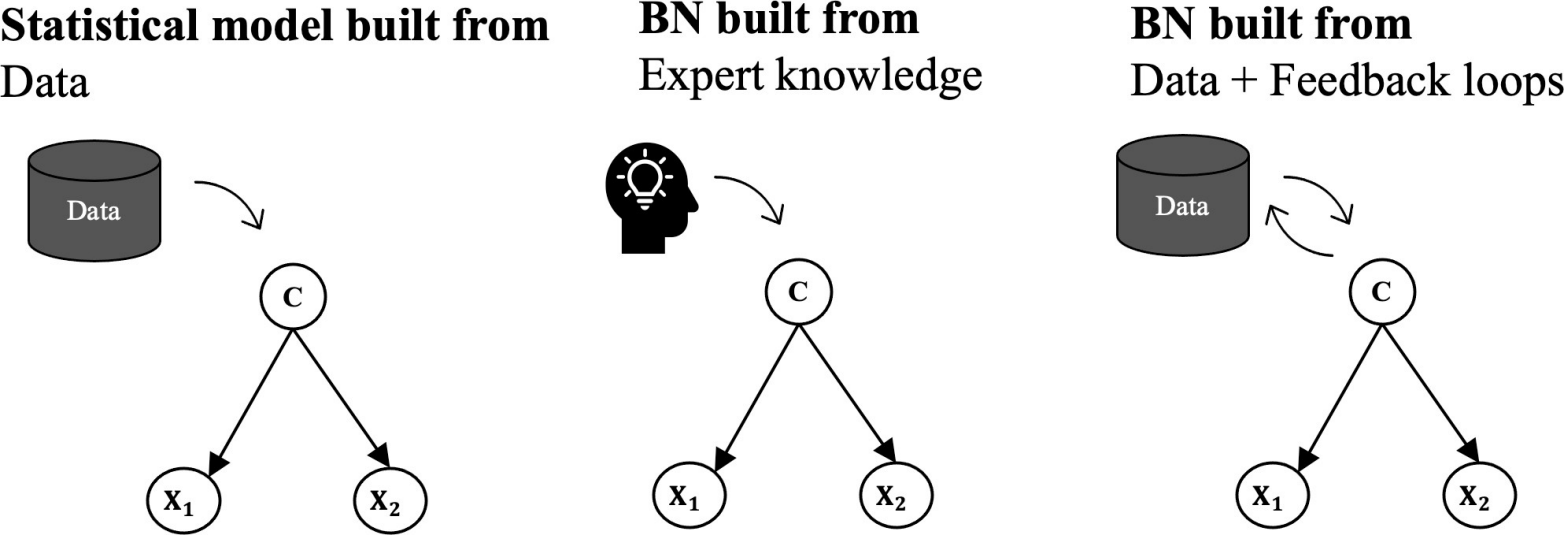
Different general approaches for building Bayesian networks.

This methodology is gaining popularity in very different fields of application for the same reasons. Just to mention a few examples, they have been used in public health evaluation [74], for risk assessment with emerging diseases [75], for medical diagnosis [76], in the Intensive Care Unit to predict survival probabilities [77] and for the criminal profile of forest arsonists [78]. Although some previous studies have already applied BNs to address archaeological questions, they have generally relied on “expert knowledge” rather than “data knowledge”, which is our approach. Fig 2 represents the three discussed approaches to learning BNs: classical statistics, expert-based ML and data-driven ML, from left to right.

As we can see in Fig 2, BNs can be built from expert knowledge, which implies that the researcher that designs the model uses prior background and expertise to define subjectively the causal directivity among variables. Conversely, BN can be data-driven and that means that causal-effect relationships are induced formally from the dataset and expressed in probabilistic terms. The resulting model can be modified and updated when new data is available.

### Implementation

#### Exploratory data analysis

Our analysis starts studying all potential binary pairings among all variables. We define the very idea of “relationship” in terms of statistical association, and we measure it in terms of association strength in a contingency table through Cramer’s V test [79] (**CramerV** function of the *DescTools* R package [80]). In this way, we offer a preliminary scanning of the parametric space to individualise those statistical relationships between ecological features and social decisions that appear most promising, i.e., that may have the greatest predictive and/or explanatory power to understand how features of settlement location may have influenced social decisions and economic strategies, and vice versa (S3 Table). Additionally, we have represented graphically the joint distribution of those pairs of variables that we have found a large association using the **balloonplot** function of the R *gplots* package [81].

With these functions, we have rigorously tested that not all variables are necessarily related to others, nor they have the same predictive/explanatory strength. In fact, only the 3% of the binary associations explored (n = 226) had a relevant statistical strength, while 21% did not show any traces of potential explanatory value. Small strength values were the most common (57%). For instance, in our dataset, the size of the community appears to be not binary related statistically with a majority of ecological factors, such as distance to coast, precipitation, etc., nor to social strategies like fishing or not fishing. Similarly, other variables regarding social organisation, like community and domestic organisation, are also not binary related to settlement area factors including slope and intensity of annual precipitation.

**Fig 3:**
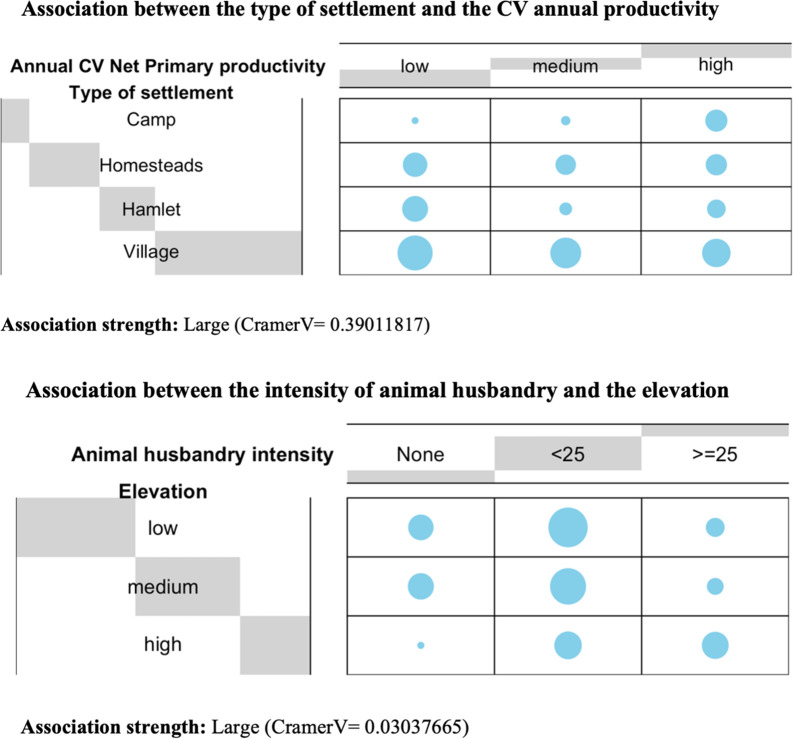
Some examples of binary associations found in the exploratory data analysis.

Conversely, we have also identified relevant statistical binary associations with high explanatory values (Fig 3). For example, the type of settlement is binary related to variations in soil net primary productivity: we can observe that bigger settlements appear located in areas where soil net primary productivity can have medium or high values, but fast never low values. On the contrary, small hamlets and homesteads settlements favour locations with low variability in soil net productivity. Another example is the relationship between animal husbandry and location, which suggests that communities decide to intensify husbandry in areas of relatively high elevation, where agriculture can be less successful.

#### Model design

The advantages of BN methods allow asking two fundamental questions to be approached inductively and probabilistically.

**Q1)** Do ecological features of settlement location and/or social organisation constrain the type and intensity of subsistence strategies? We have explored this question by analysing the probabilities of two competing hypothesis:
  - Ecological factors constrain the subsistence strategy (Input: environmental characteristics / Output: subsistence strategies),
  - Social organisation constrains the dominant form of subsistence strategy finally adopted by the community (Input: social organisation / Output: subsistence strategies).
**Q2)** Do ecological features of settlement location and/or the type and intensity of subsistence strategies constrain social organisation? We have explored this question by analysing the probabilities of three competing hypothesis:
  - Ecological factors constrain the way the community is socially organised (Input: environmental characteristics / Output: social organisation),
  - Ecological factors constrain social/economic decisions made by the community (Input: environmental characteristics / Output: social decisions),
  - The particular type of social organisation and the subsistence strategy finally adopted constrain social/economic decisions made by the community (Input: social organisation and subsistence strategies / Output: social decisions).

This definition of just a handful of restricted scenarios has allowed us to reduce the dimensionality of the parametric space and obtain meaningful results with a minimum of computational run-time. By considering only a reduced set of scenarios, we intend to group calculations into meaningful blocks. Some other scenarios could have been explored, but this is something that will be developed in forthcoming essays. We have built three structurally different networks for each scenario to determine which model has the greatest predictive and explanatory capacity:

- Models *A type* (*Binary Relevance)*: it is used to predict one output at a time, from all the input variables, so it is made up of as many BNs as output variables we have, each one with all the input and a single output variable. We have experimented with two different kinds, depending on the type of BN that is implemented:
  - Naïve Bayes (Model A-NB): It has a fixed structure, which is not learned from the data, with a directed arc from the output variable to each of the inputs, and no more.
  - Augmented Naïve Bayes (Model A-ANB). Directed arcs are allowed between the input variables, which are learned from the data.
- Model *B* consists of a single BN that contains both the input and (all) the output variables and allows them to be predicted all at the same time. Its structure is learned from the data with the only restriction that there can be no directed arcs from any input variable to any of the output variables. It is then a diagnosis-type predictive model.

**Fig 4:**
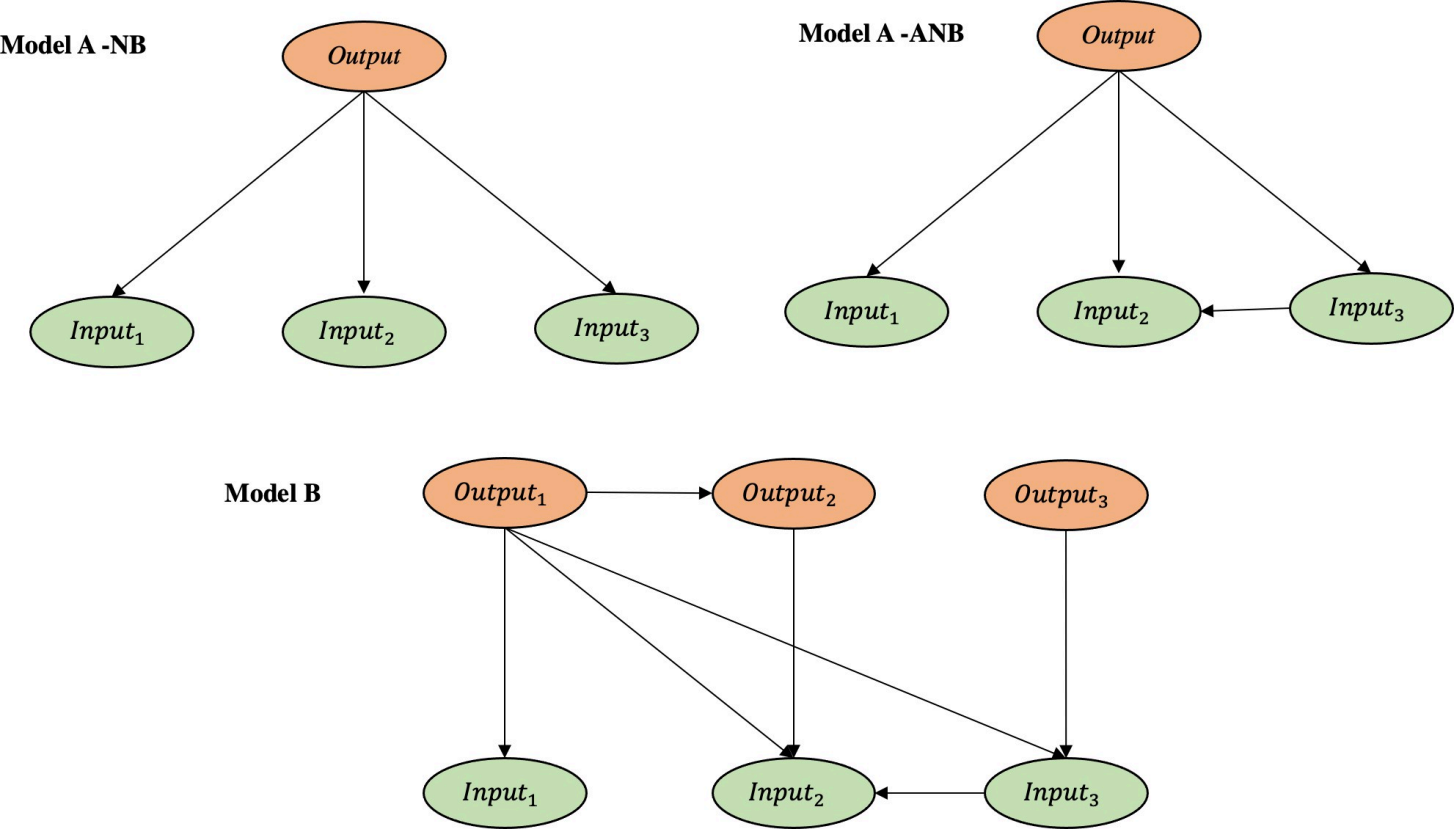
Examples of the structures of the three types of BNs in Table 3. Outputs (orange) and inputs (green).

These models, learned from the training set according to different restrictions, will entail some advantages and disadvantages for each, as specified in Table 3. See also Fig 4 for a simple example of the three types of structures illustrating the constraints on directed graphs with which they are built.

**Table 3:** Description and main characteristics of the three types of BNs.

	MODEL A: BINARY RELEVANCE (one output at a time)		MODEL B (all outputs at once)
	NB	ANB	
**Description**	Naïve Bayes (NB)	Augmented Naïve Bayes (ANB)	Unrestricted diagnosis-type BN
**Structure learning algorithm**	Fixed structure with arcs from output to inputs (black box)	Hill-climbing with AIC and BIC scores. Arcs from output to inputs mandatory (white box)	Hill climbing with AIC and BIC scores. Arcs from inputs to outputs forbidden (white box)
**Purpose**	Prediction	Prediction and explanatory (relationships between input variables)	Predictions and explanatory (relationships between input, between output, and input-output variables)
**Advantages**	Conceptual simplicity and good balance between simplicity and predictive power. Robust against unlikely evidence	Encodes the relationships between inputs, for each output separately, keeping the design relatively simple	Encodes the relationships between inputs, between outputs and between inputs and outputs. Single model in which relations between variables are not forced
**Disadvantages**	Ignores the correlations between inputs and between outputs (a different model is built for each output)	Less conceptually simple than NB, and like it, ignores the correlations between outputs (a different model is built for each output). Sensitive to unlikely evidence	More complex design than the others. Sensitive to unlikely evidence

The ultimate objective of building three different models is to compare model structures and evaluate if correlations among variables are important to consider when building a model and provide relevant information for understanding the socio-ecological systems examined.

The models have been built using the R package **bnlearn** [82], which implements structure and parameter learning. To learn the structure of Model A–ANB we used the score-based structure learning algorithm implemented in the **hc** (hill-climbing) function was used, and to make the predictions we used the R package **gRain** [83].

#### Model validation

To validate the three models in each of the five scenarios, and to be able to select which one has the highest predictive value, we have used statistical accuracy measures as a performance metric using a *k*-fold cross-validation procedure with *k* = 5. In each scenario, the dataset formed by the input variables and the output variable(s) was randomly divided into five similar folds, using four of them as a training subset to learn the model and the fifth as a test set to make predictions to evaluate the predictive power of the model by calculating its accuracy. The number of outputs for each fold depended on whether the Model was A or B. This process has been repeated *k* times, each time changing the test set training and, consequently, the training set. In this way, for each scenario and model, we obtain *k* = 5 estimates of its statistical accuracy.

First, we have compared Model A-NB with Model A-ANB. A standard statistical hypothesis test has been used based on the two samples of paired values of their accuracies. To decide whether to use the parametric paired *t*-test, or the non-parametric paired Wilcoxon signed-rank test, we first performed a Shapiro-Wilk goodness-of-fit normality test for the difference. In case of models *A*, we have privileged those with the greatest predictive capacity, to be compared with the relative Model *B* to make the best possible prediction.

Once the model with the highest accuracy is selected, the *strength* of the probabilistic relationships between the model variables expressed by the arcs of the BN is quantified through the function **arc.strength** (implemented in the *bnlearn* R package), producing a result in form of a *p*-value for a conditional independence test: the lower the *p*-value, the stronger the relationship. On the other hand, Model B has been always used for its explanatory habilities, since it is the only network typology that allows correlations between inputs and inputs and between outputs and outputs. Results of the validation process are depicted in S4 Table.

## Results

As we already knew from our initial exploratory analysis, not all variables -ecological and social/economic- have any explanatory contribution on the other. Values of output variables are hardly predictable from input variables. This result does not go in line with the prior traditional hypothesis that implies that human behaviour is necessary fitted to local ecological conditions. Our results (S5 Table) suggest that the relationship between human action and landscape is far more complex than that: different behaviours can be practised, and different social decisions can be made at different ecological, climatic, and topographical contexts.

### The influence of ecological factors on the subsistence strategies adopted

When we assume the independence between the inputs conditioned to the values of the output (Model A. Naïve-Bayes learning algorithm), our investigation suggests that ecological conditions effectively constrain hunting. That is to say, the slope degree, the distance to the coast, the monthly mean precipitation and soil productivity (average and variation) of the site catchment area have a high impact on the variations of hunting relative predominance among alternative ways of acquiring percentage of subsistence.

Gathering is also constrained by landscape feature, most notably by soil productivity variation. Fishing is mostly affected by the distance to the coast (which was expectable). Therefore, fishing would not be affected by the natural setting but it would be a direct consequence of the internal dynamics of the community: the decision about where to settle [84, 85]. This result goes in line with the study conducted by Ahedo et al. (2021) in which they identified fishing with the role of risk-mitigation function that small-scale farming communities adopt in times of scarcity [18].

The comparatively high impact of environmental conditions on the relative predominance of hunting and gathering contrasts with the low relative importance of most landscape factors on the predominance of agropastoral strategies. In this case the impact of environment seems to have had a less conspicuous role. In our results, animal husbandry is only associated with monthly mean precipitation around the settlement area. This situation can be associated with the fact that small-scale communities practising herding may practice seasonal vertical mobility to maximise herd production and survivorship in communities with mixed economies. Generally, during the late spring and early autumn animals are moved in the mountain wherereas winters are located in the lowlands plains. This practice was probably already present in the early Neolithic, as suggested in [86, 87], and there is an important corpus of ethnographical references [88, 89]. Agriculture is only related with the predominant natural vegetation around the area in which the settlement is located. It does not mean that environmental factors had no causal impact on variations of agropastoralism relative to other subsistence way, but only in the case of hunting, and secondary in gathering, the environmental would have had decisive impact.

This is a specially significative result because there is a long-standing tradition in archaeological site settlement studies of placing considerable importance on the natural setting to predict the placement of farming areas [90–93]. The underlying assumption is that by modelling the most suitable landscape for productive economic strategies, the most probable settlement and occupation locations can be predicted. However, recently, some studies have emphasised the lack of direct and linear correlation between farming and the ecological characteristics of the settled area. For instance, a recent study of Vidal-Cordasco and Nuevo-López (2021) evidences that early Neolithic communities in Iberian Peninsula had far wider ecologically diverse niches than Mesolithic populations practicing hunting and gathering, much more adapted to local recources [94]. When expanding the production area, more ecological and landscape diversity enter into the catchment area, and the relevance of local features diminishes.

In our dataset, soil natural productivity has only an impact on hunting and gathering. It contradicts traditional hypotheses suggesting that a prior high soil productivity is paramount for farming [95, 96]. Our analysis suggests the priority of social factors in taking the decision about where to settle the farm, rather on the perceived characteristics of the area. Population mobility is a social decision and it may be affected by the local possibilities for increasing labour investment, the impossibility of increasing technology efficiency or the implicit risks in the challenge of modifying the group internal organisation–social relations of production. Therefore, we can expect farming be practised even where soils may have less productivity. This result demonstrates the importance of human agency and intentionality on modifying the environment even when more suitable location -in this case, more productive soils- would have been available.

Our study also asserts that the total independence of ecological, climatic, and topographical factors among them is a hardly defendable assumption. Temperature average and temperature variation are correlated in most cases, in the same way as in the case of precipitation and soil productivity. Settlement elevation and slope are correlated in many cases. We have used the Augmented Naïve Bayes algorithm to build the interrelations among all possible ecological/climatic/topographical inputs on each kind of subsistence strategy. This analysis suggests that distance to the coast, temperature and precipitation are not totally independent among them. The same can be defined for slope and elevation, and soil productivity average and variation. The kind of landscape is mostly independent from the rest factors, although some dependence can be proved with average precipitation and/or soil productivity. In the same way, precipitation and soil productivity seem to be indirectly related will be not totally independent.

Considering these dependencies among environmental factors, the accuracy of social and economic predictions critically diminishes, because non-linear relationships affect probable consequences of human prior knowledge about the area they may settle. This result suggests the low reliability of traditional hypothesis suggesting direct and linear landscape determinism.

**Fig 5:**
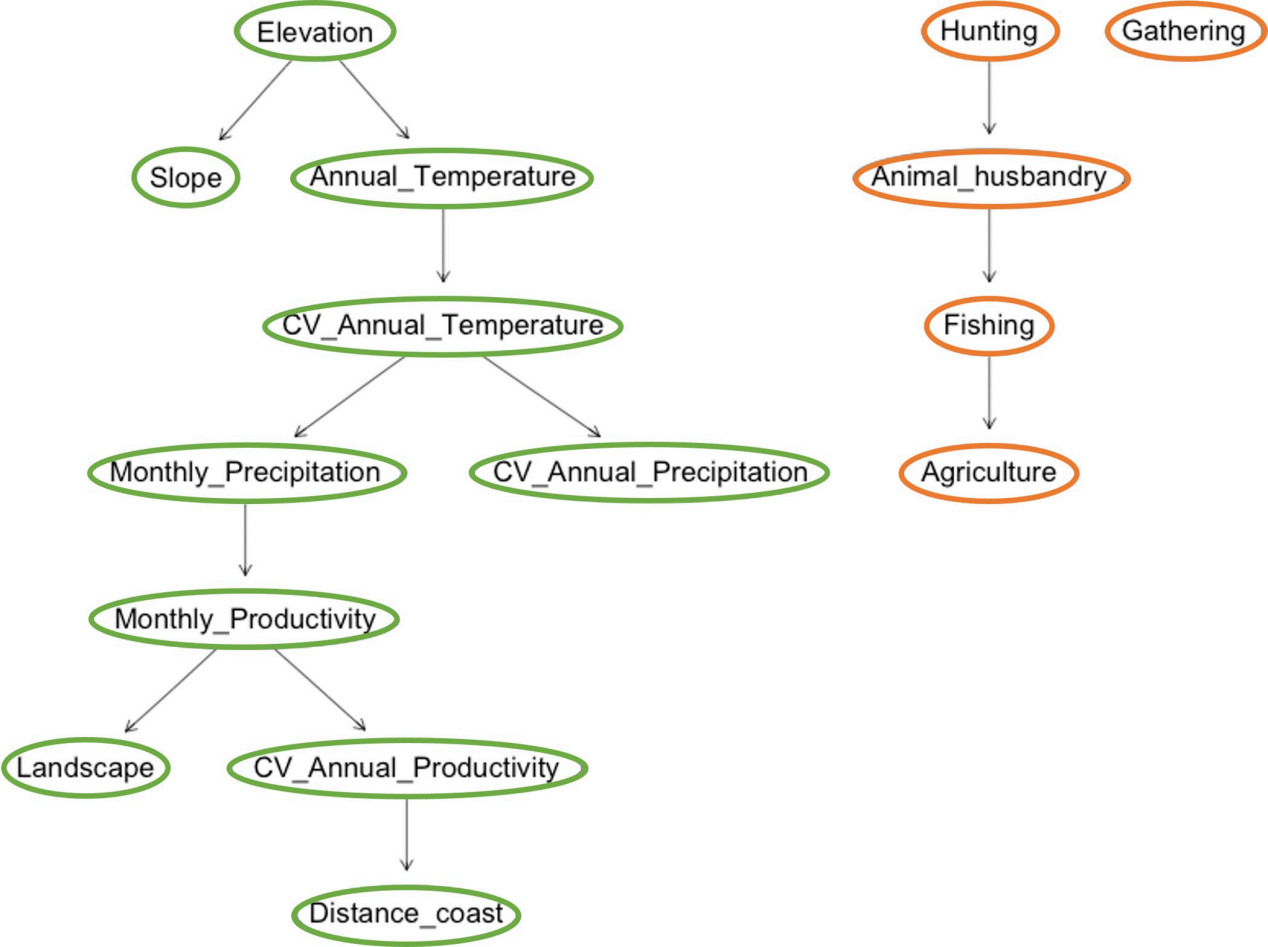
Final Model B exploring the influence of the environment on subsistence strategies. In this scenario we predicted the correlations among the ecological characteristics (inputs, green) and the subsistence strategies (outputs, orange).

Up to now we have worked with single outputs. We have not yet considered the obvious non-independence between hunting, gathering, fishing, agriculture, and husbandry (Fig 5). After all, what we are considering is the percentage of total subsistence a human group decides to acquire using different alternative strategies. It can be observed that environmental characteristics are highly interrelated as it would be expected, however, what is surprising is how economic strategies are linked. Gathering appears to be not directly linked to the rest of strategies, probably because it appears to be as a supplementary activity to increase the chances of survival rather than an independent strategy in itself. Animal husbandry plays an intermediate role: it has some indirect relation to hunting and fishing. And all of them appear to be usualy subsidiary to agriculture. The preference for fishing is related to the preference for animal husbandry (and indirectly to hunting and gathering) because of the high incidence of distance to the coast. Obviously, settlements with highest proportion of subsistence acquired by fishing are those the nearest to the coast. Our results also indicate a non-negligible influence of distance to the coast to the preference for animal husbandry and agriculture.

### The influence of ecological factors on the way the community is socially organised

**Fig 6:**
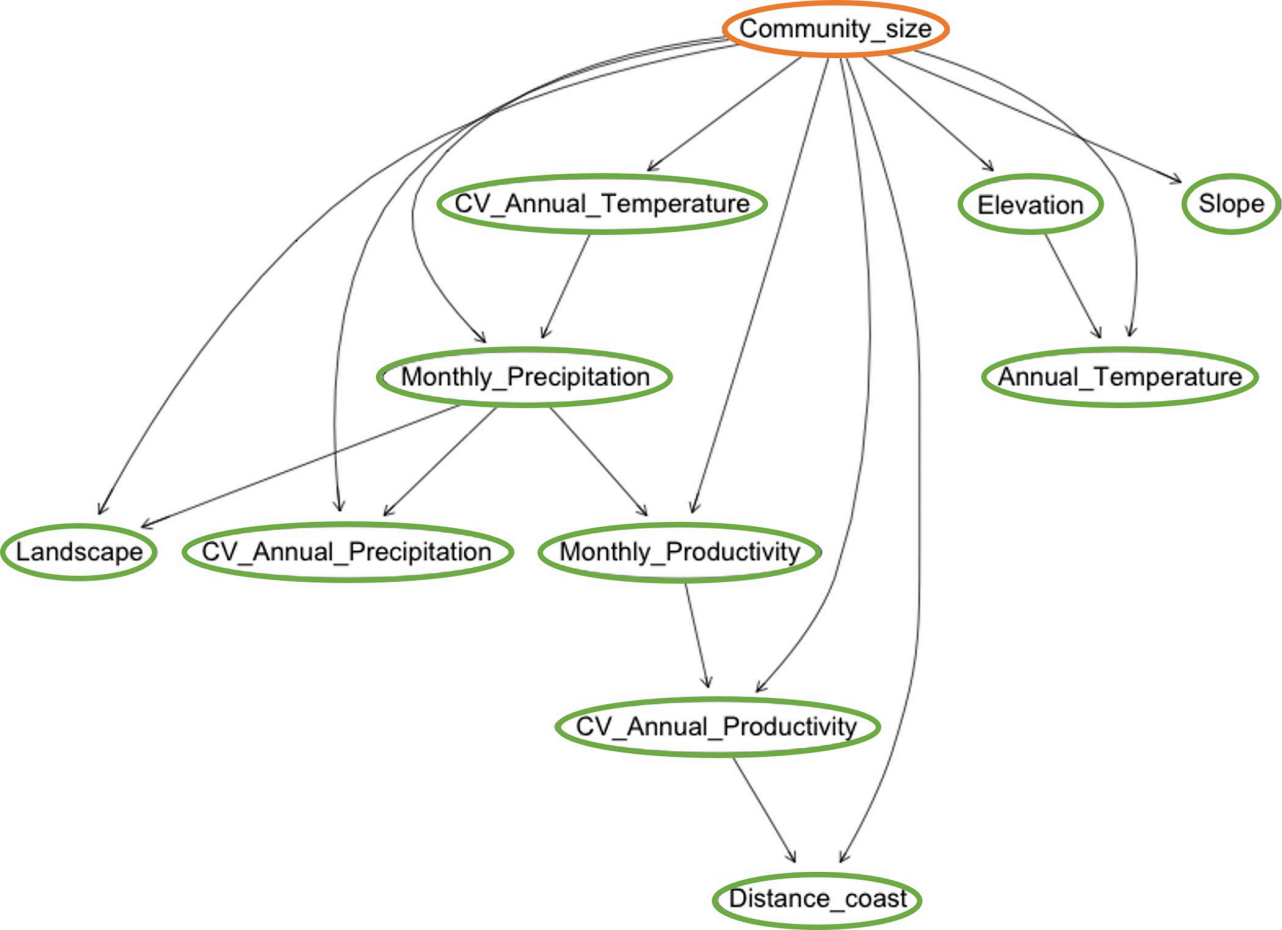
Bayesian Networks of Model A-ANB exploring the relationship between the environment and the social organisation. Dependence between ecological variables (inputs, green) and the size of community (output, orange). The relationship among the amount of inhabitants and the temperature and distance to coast is represented and it can also be observed the high dependence among the different ecological variables.

The associations found in the two Models A are almost identical, which evidences the strength of statistically induced relationships. Only the size of community, and household and community organisation are statistically correlated with some environmental factor. The size of community appears to be constrained by the annual mean and variation of temperature (Fig 6).

Our model predicts a higher correlation of community organisation with average and mean annual temperature, at also with distance to the coast. On the other hand, household organisation is dependant on the elevation of settlement area, annual temperature and the variance of annual soil productivity.

It is important to note that the dependencies among ecological, climatic and topographical factors, as discovered by the machine learning algorithm, do depend on the output. Therefore, dependencies are slightly different than those detected in the case of the influence of landscape on subsistence strategy. In general, we still observe that climatic factors are correlated between them. But now, topographical factors (elevation, slope) appear to be independent between them, probably because it is the factor with less relevance to predict social organisation.

The lack of dependencies between social organisation categories and attributes, explain the lack of accuracy in Model B predictions. This lack of dependencies is analysed in detail in later.

### The influence of ecological factors on social/economic decisions made by the community

Only significant relationships were found when it was assumed the independence of ecological, climatic, and topographical features. Storage is related to the elevation and slope in which the settlement is located, whereas the variation of soil productivity determines the intensity of exchange in-settlement.

**Fig 7:**
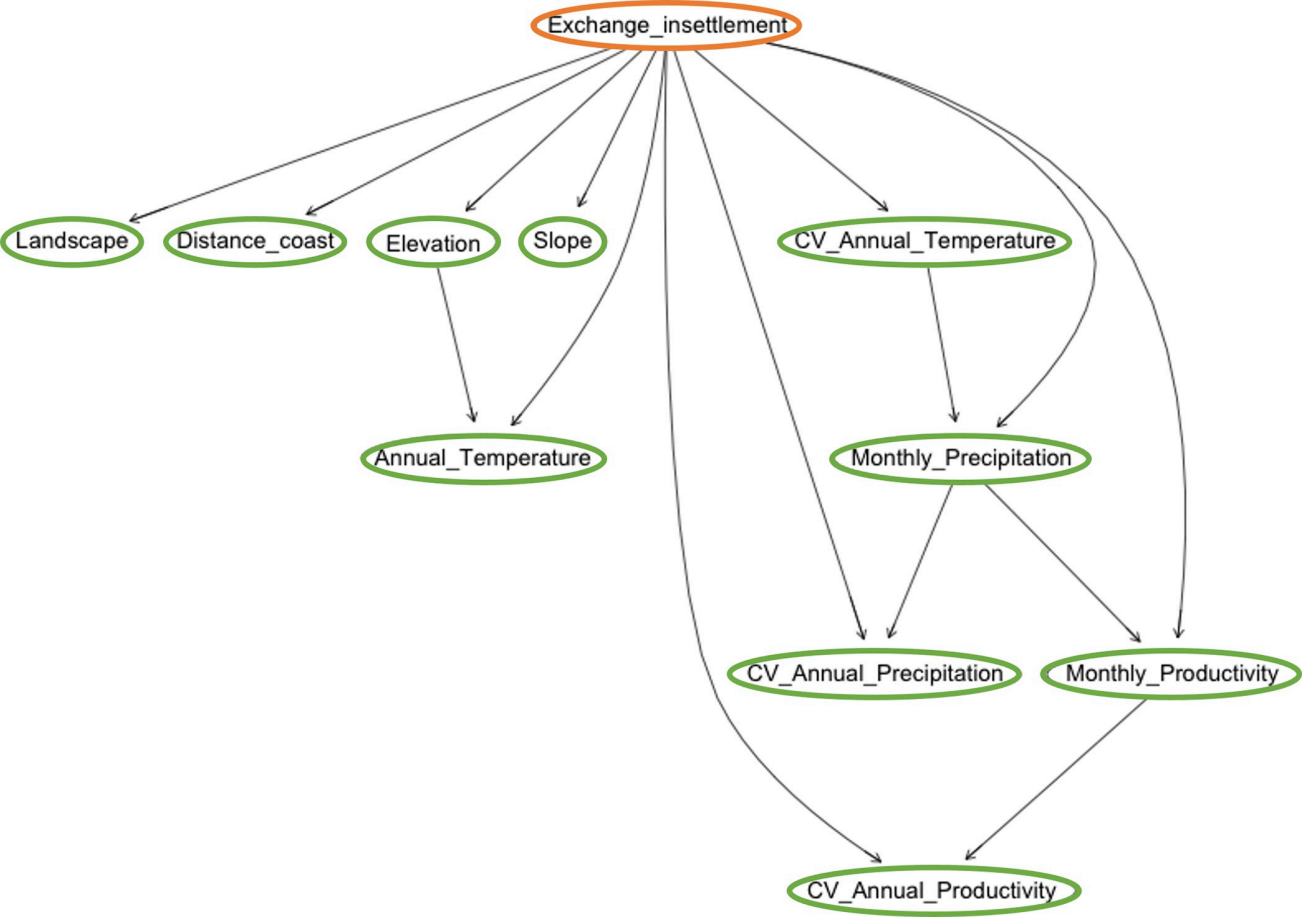
Model A-ANB investigating the relationship between the environment and social decisions. Dependence between ecological variables (inputs, green) and exchange in-settlement (output, orange).

Dependencies between ecological/climatic and topographical factors are very similar as those obtained in precedent scenarios. Their differences are not meaningful as we can observe in Fig 7.

**Fig 8:**
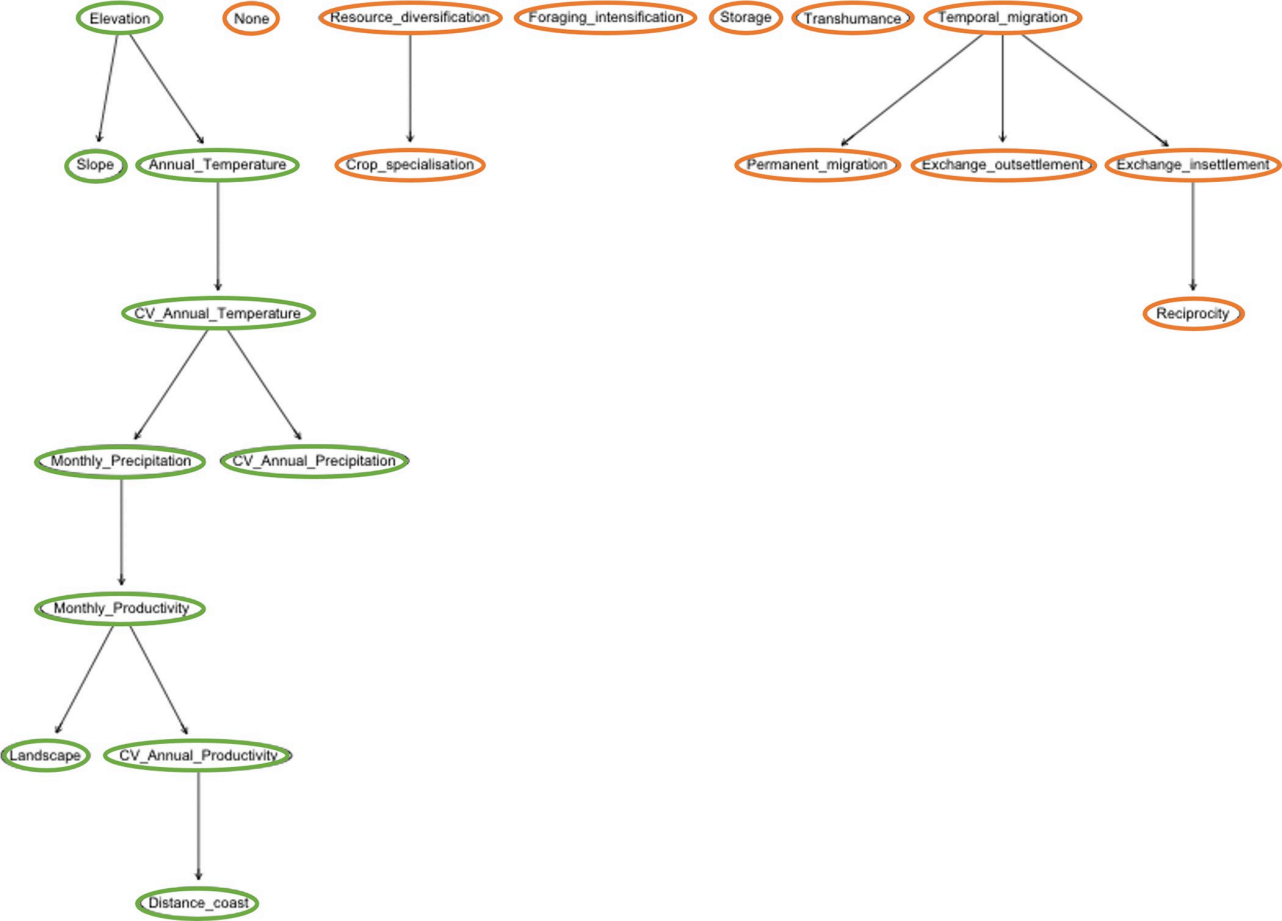
Model B representing the relationship between the environment and social decisions. This model structure is the one with the highest accuracy for this scenario and allows us to observe how strategies that imply movement (e.g., migration, exchange, etc.) are closely related.

Our Model B-type analysis shows the interrelationships among the different variables expressing social decisions (Fig 8). Temporal migration plays a major role on defining other social practices such as exchange within the community and between communities and permanent migration. Interestingly, reciprocity is related to exchange within the community, which is expectable. Resource diversification and crop specialisation appear to be related, probably because they are only decided in a minority of cases, as exceptions to the rule.

### The influence of variations in social organisation on the dominant form of subsistence strategy

**Fig 9:**
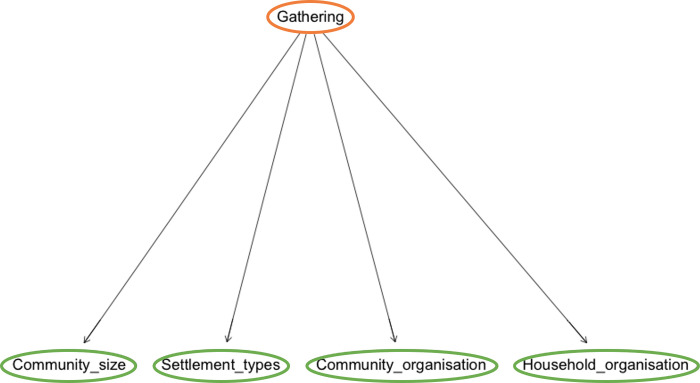
Model A-ANB exploring the relationship between social organisation and subsistence strategies. Dependence between social organisation (inputs, green) and gathering (output, orange). The absence of relationship among input variables is present in all the BNs produced for all the outputs examined.

We have only found some degrees of predictability between the mean size of the community and the type of settlement when predominant activity is agriculture, occasionally reinforced by gathering. Besides that, no other relationship appear in our dataset. The graph generated by the algorithm Augmented Naïve shows the lack of interrelationships between possible variations in social organisation and possible forms of predominant subsistence strategy (Fig 9).

Those results are obvious if we consider that the higher the number of people in the community, the more diverse will be its structure and organisation, and the greater its dependence to subsistence strategies that may generate greater volumes of subsistence. On the opposite, the lesser the size of the human group, the more efficient will be generalised gathering activities. This contradicts prior studies that have argued the lack of association between the type of settlement and the size of the community [97]. We have not found any significant impact of the forms of social organisation on the relative proportion of subsistence acquired through hunting, husbandry or fishing activities.

In most cases, the relative proportion of subsistence acquired using different strategies seems to be independent on the different possible ways of socially organising the community (size of community, settlement structure and diversity, family organisation, kinship ties, etc.). In our dataset we find instances of different forms of social organisation associated to any subsistence strategy.

### The influence of social organisation and predominant subsistence strategies on social/economic decisions made by the community

In the previous scenario we have concluded that the relative proportion of subsistence acquired by different communities and households seems to be independent of their respective social organisation. We then may expect that social organisation *and* subsistence strategy may have low incidence to explain the social and economic decisions a community may adopt in face of scarcity. In this scenario, we can observe that community organisation is related to resource diversification, which supports our prior hypothesis concerning the ability of agropastoral communities to modify and transform their niche by investing more labour and expanding the range of cultivated resources. Conversely, the specialisation on one crop as a risk-management strategy, is linked to the type of settlement and animal husbandry. The reason of this binary association between crop specialisation and animal husbandry can be explained as a negative relation, since when communities intensify and focus their production on one crop, they would reduce their herding activity. The type of settlement is related to the strategy of crop specialisation as this strategy may only be feasible in villages or permanent settlements and it is also linked to permanent migration.

## Discussion

Our analysis shows the intrinsic non-linear and non-monotone nature of the relationships between ecology, social behaviour, and economic strategies. We are not the only ones arguing for the complex and non-linear nature of the relationship between the environment and people decisions regarding subsistence and survival. In this study, however, we have partially tested how communalities between apparently different small-scale farming societies emerge when we formalise some of the environmental factors that may have affected social decisions. To address the problem of the variable predominance of alternative subsistence strategies in different social, economic, and ecological contexts, we have analysed how ecological features of settlement location and/or social organisation may have constrained the type and intensity of subsistence strategies. Results show that some landscape characteristics of the settlement area may influence indirectly the type of subsistence strategy that the community would have practised. For example, in our dataset, in an environment characterised by (1) a landscape of grassland, (2) with low and stable productivity soils, (3) located at a long distance from the coast, (4) at high elevation and (5) with steep slopes, (6) with low average temperatures but highly variable annually, (7) high average precipitation and highly variable annually, we should expect that not any human group would practise gathering or fishing. On the other hand, in an environment characterised by: (1) a landscape of forest, (2) with low and highly variable productive soils, (3) located at a short distance from the coast, (4) at medium elevation and (5) slope, (6) with medium average temperatures but highly variable annually, (7) low average precipitation and highly variable annually, we should expect that communities would base their diet on the consumption of farming products obtained through agriculture and husbandry. Therefore, different strategies are more probable in specific landscapes than others.

Our results indicate that hunting is the economic strategy more related to the local landscape conditions. The relatively low impact natural conditions had on the placement of farming settlements could be a consequence of the nature of these subsistence activities, that can be practised even when local conditions do not allow successful hunting. The hypothesis has been tested comparing the diverse forms of social organisation that different small-scale farming communities may adopt. Our results suggests that the type of settlement and the number of inhabitants play a major role in most social decisions with economic relevance. For example, when a small human community of less than 200 people live at a temporal camp, with a social organisation based on small households and clans, it is expected they adopt a subsistence strategy based on animal husbandry, complemented with hunting, and gathering. However, if the same group lived in homesteads instead of camp, farming would be the more probable source of food for them. It is significative that only a modification in the input (from temporal camp to permanent isolated homestead) brings about a so important modification on prediction.

These results demonstrate the importance of considering the type of settlement and the size of population for predicting the most probable location of the farming settlements; local environmental features would be more relevant for predicting the placement of foraging communities. Importantly, social decisions that can be adopted in face of scarcity seem to be too variable and independent of local conditions and social organisation. This result is very relevant to understand socio-ecological dynamics: human groups can build different kinds of social organisation independently to the local characteristics of their landscape.

Our investigation has also allowed to predict how *both* the environmental characteristics and the type of subsistence strategies influence significatively the way that community can be socially and economically organised. This study highlights the co-evolutionary process in the history of socially induced environmental change and small-scale farming communities social and economic decisions towards their settlement location, economic behaviour and social preferences. In contrast to social groups relying only on hunting, gathering or fishing, human communities with mixed farming economies were more diverse, and therefore individual factors constrained fewer particular forms of living and working. More variables, and not only landscape and environment, should be considered to understand how survival was possible thousands of years ago. For example, our model suggests that animal husbandry could not be limited to a singular niche, but herders could move their flocks seasonally and, therefore, adapt to many different environmental circumstances. In the same line, a especially significant finding was the lack of relationship between the preponderance of agriculture and the degree of soil productivity. Our analysis suggests that in similar way than herders, farmers could modify their niche by incrementing the labour investment and, consequently, defining the most suitable type of settlement and size of community for their economic strategy. Social decisions such as diversifying the resources, migration or exchange of foodstuffs could have played a major role for managing and compensating the resource availability in the community.

The detailed knowledge of landscape conditions by the social group could allow prehistoric communities to predict the probability of success when hunting and fishing. Gathering was not only constrained by what the landscape naturally offered, but the knowledge of the size of the community has also relevant effects on it: the lesser the size of the human group, the higher the probability that some part of the total revenue came from gathering the area around the settlement.

We suggest that in prehistory, community organisation was only partially influenced by landscape and environment, in the same way as our ethnographical generalisation has proved. This also applies to social decisions in face of scarcity, which can only indirectly be related with ecological, climatic, and topographical factors. It does not mean that the environment could have no effect at all, in fact, we have observed that the social organsiation is highly related to the climate, for example. Beyond landscape influence, our results also indicate that social decisions in face of scarcity were also influenced by the kind of dominant subsistence strategy and the way the community was organised.

## Conclusions

This paper is based on observed communalities among 173 trans-historical and cross-cultural dataset of small-scale farming societies, expressed in probabilistic terms to be able of predict social behaviour from environmental and landscape features.

Although the database is comparatively small for typical Machine Learning applications, it should take into account that it is complete for the social domain of small-scale farming societies. Those are the only well documented ethnographic cases existing in the literature. The dataset could have been increased including poorly documented cases, with a lot of missing values in the final dataset. The consequences would have been poorer accuracy in generalisations and still low precision in predictions. Therefore, we can be fairly confident of the accuracy and plausibility of our main result: the nonlinearity of the particular relationship between what people do to live, and the main features characterising the environment and the landscape where people live. The particular way people acquire their subsistence cannot be predicted without considering how people organised their settlement and their social relations of production.

The nature and reliability of calculated generalisations can be used to reconstruct, partially, how people behave and took social decisions thousands of years ago. Obviously, our results depend on the reliability of the training set used for probability estimations and predictions. In any case, we are not asserting that the past was like the present, but generalisations proved to be true in a great majority of known and well documented ethnographic cases from different chronologies and geographical areas can be considered also plausible of societies having existed in other time periods with similar ecological and environmental circumstances. What we are interested in is to consider the structural relationships between society and nature. The fact that this relationship be non-linear and indirect, affected–but not determined- by human particularity, only makes the reconstruction of prehistoric ways of living more difficult. We rely on the language of probabilities, and Bayesian reasoning, to explore scenarios that were “probable” in the past, although we have not the full evidence.

Methodologically, the main result of this investigation lies on the recognition of the relevance of the independence between input factors, between output factors and between input and output factors. Assuming independence has been the traditional assumption in most socio-ecological investigations. Our analysis signals the misleadingness of this assumption, and the need of considering the way the causal influence of a factor has on another factor to be able to predict how a small farming community may have reacted locally.

Bayesian Networks, the kind of machine learning algorithm used along this paper, show their value for understanding socio-ecological systems. They are useful and versatile “white box” models that clearly describe the relationships and patterns between the variables involved in a phenomenon while providing predictions about the most probable value of an *output* variable of interest and generate new knowledge. By building three different learning algorithms, modelled to explore alternatives assumptions and scenarios (Naïve Bayes, Augmented Naïve Bayes, Unrestricted Diagnosis Networks), we have demonstrated that not all assumptions have the same predictive and explanatory potential.

With Bayesian Networks it is possible to identify a connection between what could have happened in the past and the material evidence that this action caused then and is observable in the present. However, it should be borne in mind that this study represents a preliminary investigation, since we have worked with a limited number of cases because they were the ones available in the two databases that we have consulted and that followed the requirements set. However, the number of cases should be increased to assess whether the same relationships between variables are still observed. Another aspect that we would investigate in the future is to explore our database with Gaussian or hybrid Bayesian Networks, the latter allow working with both numerical and categorical input variables, while with the Gaussian BN we can only consider continuous input variables. Standard Bayesian networks only deal with discrete/categorical variables and, as consequence, we have been forced to discretize the continuous variables, with the consequent loss of information. Likewise, working with more case studies, continuous data and conditionally Gaussian Bayesian Networks, we would hope to describe better the relationships between variables and identify nuances that with the current model have not been possible.

## Supporting information

S1 Table Relevant variables to consider for building socio-ecological models according to prior studies. (PDF)

S2 Table Variables description. * We measured the coefficient of variation (CV) to explore the ratio of the standard deviation to the mean. We used the mean and variance, both specified in D-PLACE datavase [60]. ** Monographies of each society are specified in S2 Dataset and were extracted from eHRAF dataset [61]. *** *Homesteads*: isolated domestic unit; *Camp*: temporary location of huts or other structures where households live collectively during that period; *Hamlet*: formed by few several homesteads, it can be both dispersed and clustered; *Village*: like the hamlet, it is composed by several homesteads and it can also be both dispersed and clustered, but the village has more homesteads. It is the largest type of settlement in this classification.
(XLSX)

S3 Table Summary of the relevant relationships found. We followed [84] guidelines for interpreting relevant relationships of Cramer’s V test: **k = 2:** Small (0.10–0.30), Medium (0.30–0.50), Large (>0.50); **k = 3:** Small (0.07–0.20), Medium (0.20–0.35), Large (>0.35); **k = 4:** Small (0.06–0.17), Medium (0.17–0.29), Large (>0.29).
(XLSX)

S4 Table Summary of the results of the statistical tests of hypotheses for the comparison of the predictive models, in each scenario. Only significant p-values are depicted in the table. Empty cells correspond to statistically non-significant probabilities. “Model 1 > Model 2” means that the alternative hypothesis (which is accepted if the *p*-value is < 0.05) is that the mean accuracy is greater for the Model 1 than for the Model 2. *P*-values are classified as follows: 0.05–0.01 = *; 0.01–0.001 = **; <0.001 = ***. In Model B, significant relationships among inputs are not represented (only among outputs) to avoid noise from variables that we do not predict and are expectable (i.e., the relationship among environmental characteristics).
(XLSX)

S5 Table Strength of the relationships of all scenarios. *P*-values are classified as follows: 0.1–0.05 = ·; 0.05–0.01 = *; 0.01–0.001 = **; <0.001 = ***.
(XLSX)

S1 Dataset Dataset employed in this study. A total of 174 case studies and 30 different variables are considered.
(XLSX)

S2 Dataset Consulted dataset for identifying the adaptive strategies of each case study. (XLSX)

S1 File (DOCX)
