# Supplementary material for: Exploring the role of ecology and social organisation in agropastoral societies: A Bayesian network approach
Source: PLoS One. 2022 Oct 26;17(10):e0276088. doi: 10.1371/journal.pone.0276088 (PMC9605033; doi:10.1371/journal.pone.0276088)
Supplement: S1 Table — (PDF) [file pone.0276088.s001.pdf]

Relevant data for modelling socio-ecological systems	References of previous studies
Type, availability, and abundance of resources in the environment	[1-9]
Intensity that the subsistence strategy is practised	[2, 10-15]
Population size	[2, 16-17]
Productivity of different economic resources	[2, 18-31]
Settlement location	[32-33]
Soil characteristics	[31, 33, 34-39]
Weather characteristics	[34, 40-43]
Household organisation	[44-46]
Community organisation	[47-52]
Environmental and soil productivity degradation	[53-56]
Labour organisation	[57]
Labour costs	[58-61]
Culture	[62]
Social decisions (e.g., exchange, migration, storage, diversification, intensification, etc)	[63-68]
