# Supplementary material for: Exploring the role of ecology and social organisation in agropastoral societies: A Bayesian network approach
Source: PLoS One. 2022 Oct 26;17(10):e0276088. doi: 10.1371/journal.pone.0276088 (PMC9605033; doi:10.1371/journal.pone.0276088)
Supplement: S1 File — (DOCX) [file pone.0276088.s008.docx]

**References Supporting Information**

1. Carles AB. Sheep production in the tropics. 1983.
2. Rindos D. The Origins of Agriculture: An Evolutionary Perspective. Academic Press. 1984.
3. Lubbering JM, Stuth JW, Mungall EC, Sheffield WJ. An approach for strategic planning of stocking rates for exotic and native ungulates. Applied Animal Behaviour Science. 1991 1;29(1-4):483-8.
4. Nablusi H, Ali JM, Abu Nahleh J. Amman Sheep and Goat Management Systems in Jordan: Traditional and Feedlot, a Case Study. Task Force Documents. 1993.
5. Gulelat W. Household herd size among Pastoralists in relation to overstocking and rangeland degradation. International institute for geo-information science and earth observation, Enschede, The Netherlands. 2002.
6. Degen AA. Sheep and goat milk in pastoral societies. Small Ruminant Research. 2007;68(1-2):7-19.
7. Wilkinson TJ, Rayne L, Jotheri J. Hydraulic landscapes in Mesopotamia: The role of human niche construction. Water History. 2015;7(4):397-418.
8. Baum T, Nendel C, Jacomet S, Colobran M, Ebersbach R. “Slash and burn” or “weed and manure”? A modelling approach to explore hypotheses of late Neolithic crop cultivation in pre-alpine wetland sites. Veget Hist Archaeobot. 2016;25(6):611–27.
9. Baum T. Simulating Land Use of Prehistoric Wetland Settlements: Did Excessive Resource Use Necessitate a Highly Dynamic Settlement System? In: Barceló JA, Del Castillo F, editors. Simulating Prehistoric and Ancient Worlds. Cham: Springer International Publishing; 2016;255–279.
10. Reynolds PJ, Hedge RW. The Butser Ancient Farm Earthworks Research Project: A First Analysis of the Data and a Description and Review of the Methodology. Butser Ancient Farm; 1999.
11. Wilkinson TJ. (1997). Environmental fluctuations, agricultural production and collapse: a view from Bronze Age Upper Mesopotamia. In Third millennium BC climate change and Old World collapse (pp. 67-106). Springer, Berlin, Heidelberg.
12. Van Driel G. The Mesopotamian north: Land use, an attempt. Rainfall and agriculture in northern Mesopotamia. 2000:265-99.
13. McClure SB, Jochim MA, Barton CM. Human behavioral ecology, domestic animals, and land use during the transition to agriculture in Valencia, eastern Spain. Behavioral ecology and the transition to agriculture. 2006,2:197-216.
14. Hajnalová M, Dreslerova D. Ethnobotany of einkorn and emmer in Romania and Slovakia: towards interpretation of archaeological evidence. Památky archeologické. 2010;169-202.
15. Wilkinson TJ, Rayne L, Jotheri J. Hydraulic landscapes in Mesopotamia: The role of human niche construction. Water History. 2015;7(4):397-418.
16. Ortman SG, Varien MD, Gripp TL. Empirical Bayesian methods for archaeological survey data: An application from the Mesa Verde region. American Antiquity. 2007;72(2):241-72.
17. Kohler TA, Reese KM. Long and spatially variable Neolithic demographic transition in the North American Southwest. Proceedings of the National Academy of Sciences. 2014;111(28):10101-6.
18. Sauer CO. Agricultural origins and dispersals. The American Geographical Society; 1952.
19. Braidwood RJ, Howe B. Prehistoric Investigations in Iraqi Kurdistan. Studies in ancient oriental civilization. 1960;31.
20. Carter DL, Berg RD, Sanders BJ. The effect of furrow irrigation erosion on crop productivity. Soil Science Society of America Journal. 1985;49(1):207-11.
21. Christensen LA, McElyea DE. Toward a general method of estimating productivity-soil depth response relationships. Journal of Soil and Water Conservation. 1988;43(2):199-202.
22. Harris DR. An evolutionary continuum of people–plant interaction. InThe Emergence of Agriculture 2020 (pp. 26-44). Routledge.
23. Gross E, Jacomet S, Schibler J. Stand und Ziele der wirtschaftsarchäologischen Forschung an neolithischen Ufer-und Inselsiedlungen im unteren Zürichseeraum (Kt. Zürich, Schweiz). Beiträge zur Archäozoologie, Archäologie, Anthropologie, Geologie und Paläotologie (Festschrift HR Stampfli). 1990:77-100.
24. Araus JL, Amaro T, Zuhair Y, Nachit MM. Effect of leaf structure and water status on carbon isotope discrimination in field‐grown durum wheat. Plant, Cell & Environment. 1997;20(12):1484-94.
25. Araus JL, Amaro T, Voltas J, Nakkoul H, Nachit MM. Chlorophyll fluorescence as a selection criterion for grain yield in durum wheat under Mediterranean conditions. Field Crops Research. 1998;55(3):209-23.
26. Romagosa I, Slafer GA, Araus JL. Durum wheat and barley yields in antiquity estimated from 13C discrimination of archaeological grains: a case study from the Western Mediterranean Basin. Functional Plant Biology. 1999;26(4):345-52.
27. Sadras VO, Calvino PA. Quantification of grain yield response to soil depth in soybean, maize, sunflower, and wheat. Agronomy Journal. 2001;93(3):577-83.
28. Barzegar AR, Yousefi A, Daryashenas A. The effect of addition of different amounts and types of organic materials on soil physical properties and yield of wheat. Plant and soil. 2002;247(2):295-301.
29. Quiroga A, Funaro D, Noellemeyer E, Peinemann N. Barley yield response to soil organic matter and texture in the Pampas of Argentina. Soil and Tillage Research. 2006;90(1-2):63-8.
30. Pswarayi A, Van Eeuwijk FA, Ceccarelli S, Grando S, Comadran J, Russell JR, Francia E, Pecchioni N, Li Destri O, Akar T, Al‐Yassin A. Barley adaptation and improvement in the Mediterranean basin. Plant Breeding. 2008;127(6):554-60.
31. Baum T, Mainberger M, Taylor T, Tinner W, Hafner A, Ebersbach R. How many, how far? Quantitative models of Neolithic land use for six wetland sites on the northern Alpine forelands between 4300 and 3700 bc. Vegetation History and Archaeobotany. 2020;29(6):621-39.
32. Orians GH. On the theory of central place foraging. Analysis of ecological systems. 1979:157-77.
33. Barton CM, Ullah I, Mitasova H. Computational Modeling and Neolithic Socioecological Dynamics: A Case Study from Southwest Asia. Am antiq. 2010 Apr;75(2):364–86.
34. Cain J. Planning improvements in natural resource management. guidelines for using bayesian networks to support the planning and management of development programmes in the water sector and beyond. 2001.
35. Christiansen JH, Altaweel M. Simulation of natural and social process interactions in bronze age mesopotamian settlement systems. 69th Annual proceedings of the society for American archeology. 2004.
36. Christiansen J, Altaweel M. Understanding ancient societies: a new approach using agent-based holistic modeling. Structure and Dynamic: eJournal of Anthropological and Related Sciences. 2006;1(2).
37. Wilkinson TJ, Christiansen JH, Ur J, Widell M, Altaweel M. Urbanization within a dynamic environment: modeling Bronze Age communities in Upper Mesopotamia. American anthropologist. 2007 Mar;109(1):52-68.
38. Altaweel M. Investigating agricultural sustainability and strategies in northern Mesopotamia: results produced using a socio-ecological modeling approach. Journal of Archaeological Science. 2008 Apr 1;35(4):821-35.
39. Neteler M, Mitasova H. Open source GIS: a GRASS GIS approach. Springer Science & Business Media; 2013 Apr 18.
40. Robertson EC. Space and spatial analysis in archaeology. UNM Press; 2006.
41. Nowak MA. Five rules for the evolution of cooperation. science. 2006 Dec 8;314(5805):1560-3.
42. Kohler TA, Gumerman GJ, Reynolds RG. Simulación teórica de sociedades antiguas. Investigación y ciencia. 2005(348):66-73.
43. Miller A, Barton CM, García O, Bernabeu J. Surviving the Holocene: Human Ecological Responses to the Current Interglacial in Southern Valencia, Spain. Journal of anthropological research. 2009 Jul 1;65(2):207-20.
44. Fleuret P, Fleuret A. Fuelwood use in a peasant community: a Tanzanian case studyInternational Workshop on Energy Survey Methologies for Developing Countries. Proceedings. InInternational Workshop on Energy Survey Methodologies for Developing Countries, Jekyll Island, Ga.(EUA), 21-25 Ene 1980 (No. 18761). National Academy of Sciences, Washington, DC (EUA).
45. Kohler TA, Van West CR, Carr EP, Langton CG. Agent-based modeling of prehistoric settlement systems in the Northern American Southwest. InProceedings of the Third International Conference on Integrating GIS and Environmental Modelling, Santa Fe, NM 1996 Jan 21.
46. Baum TG. Models of wetland settlement and associated land use in South-West Germany during the fourth millennium BC. Vegetation history and archaeobotany. 2014 May;23(1):67-80
47. Sahlins M. Stone Age. Economics. 1972:395-428.
48. Henrich J, Boyd R, Bowles S, Camerer C, Fehr E, Gintis H, McElreath R. Cooperation, reciprocity and punishment in fifteen small-scale societies. American Economic Review. 2001 May;91(2):73-8.
49. Turchin P, Korotayev A. Population density and warfare: A reconsideration. Social Evolution & History. 2006;5(2):121-58.
50. Kohler TA, Varien MD, Wright AM, Kuckelman KA. Mesa Verde Migrations: New archaeological research and computer simulation suggest why ancestral Puebloans deserted the northern Southwest United States. American Scientist. 2008 Mar 1;96(2):146-53.
51. Ebersbach R. My farmland–our livestock. Forms of subsistence farming and forms of sharing in peasant communities. The Principle of Sharing. Segregation and Construction of Social Identities at the Transition from Foragin to Farming. Berlin: Ex Oriente (Studies in early Near Eastern production, subsistence, and environment 14), 2010,159-82.
52. Apicella CL, Marlowe FW, Fowler JH, Christakis NA. Social networks and cooperation in hunter-gatherers. Nature. 2012 Jan;481(7382):497-501.
53. Rösch M, Ehrmann O, Herrmann L, Schulz E, Bogenrieder A, Goldammer JP, Hall M, Page H, Schier W. An experimental approach to Neolithic shifting cultivation. Vegetation History and Archaeobotany. 2002 Jun;11(1):143-54.
54. Zeder MA. The broad spectrum revolution at 40: resource diversity, intensification, and an alternative to optimal foraging explanations. Journal of Anthropological Archaeology. 2012,31(3), 241-264.
55. Vogt R, Kretschmer I. (2019). Archaeology and agriculture: conflicts and solutions. E&G Quaternary Science Journal, 68(1), 47-51.
56. Ehrmann O, Biester H, Bogenrieder A, Rösch M. (2014). Fifteen years of the Forchtenberg experiment—results and implications for the understanding of Neolithic land use. Vegetation history and archaeobotany, 23(1), 5-18.
57. Kennett DJ, Winterhalder B. Behavioral ecology and the transition to agriculture (Vol. 1). 2006, Univ of California Press.
58. Bettinger RL. Hunter-gatherer foraging: five simple models. ISD LLC; 2009 Dec 31.
59. Bettinger RL, Garvey R, Tushingham S. Hunter-gatherers: archaeological and evolutionary theory. Springer; 2015 Jun 30
60. Herzog NM, Goodale N. Human behavioral ecology and technological decision-making. In Handbook of Evolutionary Research in Archaeology. 2019 (pp. 295-309). Springer, Cham.
61. Nagaoka L. Human behavioral ecology and zooarchaeology. InHandbook of evolutionary research in archaeology 2019 (pp. 231-253). Springer, Cham.
62. Cohen MN. Archaeological evidence for population pressure in pre-agricultural societies. American Antiquity. 1975 Oct;40(4):471-5.
63. Winterhalder B. Gifts given, gifts taken: the behavioral ecology of nonmarket, intragroup exchange. Journal of Archaeological Research. 1997;5(2), 121-168.
64. Winterhalder B, Smith EA. Analyzing adaptive strategies: Human behavioral ecology at twenty-five. Evolutionary Anthropology. 2000;9(2), 51-72.
65. Marti-Grädel E, Deschler-Erb S, Hüster-Plogmann H, Schibier J. Early evidence of economic specialization or social differentiation: a case study from the Neolithic lake shore settlement'Arbon-Bleiche 3'(Switzerland). Behaviour Behind Bones: The Zooarchaeology of Ritual, Religion, Status and Identity, 2003,164.
66. Starkovich BM. Dietary changes during the upper Palaeolithic at Klissoura cave 1 (Prosymni), Peloponnese, Greece. Before Farming. 2009;3:1-4.
67. Nettle D. Ecological influences on human behavioural diversity: a review of recent findings. Trends in ecology & evolution. 2009 Nov 1;24(11):618-24.
68. Stiner MC, Munro ND, Sanz M. Carcass damage and digested bone from mountain lions (Felis concolor): implications for carcass persistence on landscapes as a function of prey age. Journal of Archaeological Science. 2012 Apr 1;39(4):896-907.
69. Murdock GP, Textor R, Barry H, White DR, Gray JP, Divale WT. Ethnographic Atlas. World Cultures. 1999 10:24-136 (codebook)
70. Olson DM, Dinerstein E, Wikramanayake ED, Burgess ND, Powell GV, Underwood EC, D'amico JA, Itoua I, Strand HE, Morrison JC, Loucks CJ. Terrestrial Ecoregions of the World: A New Map of Life on EarthA new global map of terrestrial ecoregions provides an innovative tool for conserving biodiversity. BioScience. 2001 Nov 1;51(11):933-8.
71. Wessel P, Smith, WHF. Global Self-consistent, Hierarchical, High-resolution Geography Database (GSHHS) v2.3.4 [Internet]. 2015. Available: https://www.ngdc.noaa.gov/mgg/shorelines/gshhs.html
72. Danielson JJ, Gesch DB. Global multi-resolution terrain elevation data 2010 (GMTED2010). Washington, DC, USA: US Department of the Interior, US Geological Survey; 2011.
73. Lima-Ribeiro MS, Varela S, González-Hernández J, de Oliveira G, Diniz-Filho JA, Terribile LC. EcoClimate: a database of climate data from multiple models for past, present, and future for macroecologists and biogeographers. Biodiversity Informatics. 2015 Aug 23;10
74. NASA. Net Primary Productivity (1 month - TERRA/MODIS) [Internet]. Available: http://neo.sci.gsfc.nasa.gov/view.php?datasetId=MOD17A2_M_PSN
75. Kirby K, Greenhill S, Forkel R. Ethnographic Atlas. Jena: Max Planck Institute for the Science of Human History. 2018. (Available online at http://dplace2.clld.org/contributions/EA, Accessed on 2022-05-02.)
76. Binford L. Constructing Frames of Reference: An Analytical Method for Archaeological Theory Building Using Hunter-gatherer and Environmental Data Sets. University of California Press. 2001.
77. Whyte MK. The status of women in preindustrial societies. Princeton University Press. 2015
78. Karalekas D. Navigating Terra Nullius: The Ababda and the Case for Indigenous Land Rights in Bir Tawil. 2020;4(2):10.
79. Abdel-Qadr M, Wendrich W, Kosc Z, Barnard H. Giving a voice to the Ababda. In: The history of the peoples of the eastern desert. ISD LLC; 2012. p. 398–415.
80. Bos-Seldenthuis, JEMF. Life and tradition of the Ababda nomads in the Egyptian desert, the junction between intangible and tangible heritage management. International Journal of intangible heritage. 2007;2:31–43.
81. Maptia. Mapta website [Internet]. [cited 2021 Mar 15]. Available from: https://maptia.com/martaprzybyl/stories/adi-tribe
82. Singh UK. Arunachal Pradesh: A study of the legal system of the Adi tribe. Har-Anand Publications in association with Vikas Pub; 2011. 105 p.
83. Singh RK, Zander KK, Kumar S, Singh A, Sheoran P, Kumar A, et al. Perceptions of climate variability and livelihood adaptations relating to gender and wealth among the Adi community of the Eastern Indian Himalayas. Applied Geography. 2017 Sep;86:41–52.
84. Singh RK, Rallen O, Padung E. Elderly Adi Women of Arunachal Pradesh: “Living Encyclopedias” and Cultural Refugia in Biodiversity Conservation of the Eastern Himalaya, India. Environmental Management. 2013 Sep;52(3):712–35.
85. Raj S. Traditional knowledge, innovation systems and democracy for sustainable tribes in Adi tribes of eastern Himalayas of north-east India. 2010;11.
86. Tangjang S, Borang A, Arunachalam A. Improving Sustenance of Small and Marginal (Adi) Farmers through Traditional Vegetable Crops in East Siang District of Arunachal Himalaya Northeast India. 2014;27(1):5.
87. Indian Tribal Heritage website [Internet]. [cited 2021 Mar 10]. Available from: https://indiantribalheritage.org/?p=30419#gsc.tab=0
88. Needham R. A structural analysis of Aimol society. Journal of the Humanities and Social Sciences of Southeast Asia. 1960;116(1):81–108.
89. Alagbam TD. An overview of the land and the Aimol People. [Department of Anthropology]: Manipur University; 2012.
90. Kammerer CA. Descent, alliance, and political order among Akha. American ethnologist. 1998;25(4):659–74.
91. Anderson EF. Ethnobotany of hill tribes of northern Thailand. I. Medicinal plants of Akha. Econ Bot. 1986 Jan;40(1):38–53.
92. Pitchayakan P. The inter-relationship of subsistence economy and population problem: a study of the Akha case in Thailand.1982;89:54.
93. Du Bois CA, Kardiner A, Oberholzer E. People Of Alor: A Social-Psychological Study Of An East Indian Island. Minneapolis: University of Minnesota Press; 1944.
94. Adams KM. Preliminary Survey of Alor. Wisconsin: Hewlett-Mellon Fund; 1989.
95. DuBois CA. The Alorese. In: Psychological Frontiers of Society. New York: Columbia University Press; 1945,101–258.
96. Lee KC, Karimova PG, Yan SY, Li YS. Resilience Assessment Workshops: A Biocultural Approach to Conservation Management of a Rural Landscape in Taiwan. Sustainability. 2020 Jan 4;12(1):408.
97. Chen YS, Liu LW. Interaction and Integration of Cultural Inheritance, Ecotourism, and Industrial Development.: Strategies, Mechanisms and Spatial Practice of The Amis Dietary Culture Transformed into Creativity Living Industry. IRSPSD International. 2017;5(3):66–78.
98. Isichei E. On Being Invisible: An Historical Perspective of the Anaguta and Their Neighbors. The International Journal of African Historical Studies. 1991;24(3):513.
99. Isichei E. Change in Anaguta Traditional Religion. 1991;25.
100. Bacon CRK. The Anuak. Sudan Notes and Records. 1922.5(3):113–29.
101. Lienhardt G. Anuak Village Headmen. I. Africa. 1957;27(4):341–55.
102. Lienhardt G. Anuak village headmen: II’. Africa. 1958;23–36.
103. Wall LL. Anuak Politics, Ecology, and the Origins of Shilluk Kingship. Ethnology. 1976 Apr;15(2):151.
104. Mao AA, Odyuo N. Traditional fermented foods of the Naga tribes of Northeastern, India. 2007;6(1):5.
105. Imchen A, Joglekar PP. Traditional Fishing Practices among the Ao Nagas: A Case Study of Mangmetong Village, Nagaland. 2017:11.
106. Mills JP. Certain Aspects of Naga Culture. The Journal of the Royal Anthropological Institute of Great Britain and Ireland. 1926;56:27.
107. Waller R. Ecology, Migration, and Expansion in East Africa. African Affairs. 1985 Jul;84(336):347–70.
108. Tabeau PA. Tabeau’s Narrative of Loisel’s Expedition to the Upper Missouri. University of Oklahoma Press; 1939.
109. Murie JR. Ceremonies of the Pawnee. University of Nebraska Press; 1989.
110. Holder P. Social Stratification among the Arikara. Ethnohistory. 1958;5(3):210.
111. Aquino TVD, Iglesias MP. Zoneamento ecológico-econômico do Acre. Terras e populações indígenas, Rio Branco. Instituto do Meio-Ambiente; 1999.
112. Espinosa O. Los Asháninka: Guerreros en una historia de violencia. America indigena. 1993;53(4):45–60.
113. Peralta PA, Kainer KA. Market Integration and Livelihood Systems: A Comparative Case of Three Asháninka Villages in the Peruvian Amazon. Journal of Sustainable Forestry. 2008 Sep 2;27(1–2):145–71.
114. Li YY, Shih L, Yuan CR, Yang FF. The Atayal of Nan-ao, Taipei: The Institute of Ethnology. Academia Sinica; 1963.
115. Mori U. Ethnography of Taiwanese Savage Tribes. The Institute of Ethnology: Academia Sinica; 1917.
116. Wang M. The Reinvention of Ethnicity and Culture: A Comparative Study on the Atayal and the Truku in Taiwan.2006 :44.
117. Czekanowski J. Research in the Nile-Congo Region. Leipzig: Klinkhardt und Biermann; 1924.
118. Reynolds H. Notes on the Azandé tribe of the Congo. African Affairs. 1904 Apr;3(XI):238–46.
119. Phillips UB. Nilotics and Azande. New York: The Trustees; 1930.
120. Evans-Pritchard EE. The Azande. Oxford: Clarendon Press; 1971.
121. Lévi-Strauss C. The Tribes of the Upper Xingu River. In: Handbook of South American Indians. Washington: Bureau of American Ethnology Bulletin; 1950;143(3):321–48.
122. Oberg K. The Bacairí of Northern Matto Grosso. Southwestern Journal of Anthropology. 1948 Oct;4(3):305–19.
123. Petrullo V. Primitive Peoples of Matto Grosso. Philadelphia Museum Journal. 1932;23(2):83–173.
124. Opler M, Singh R. Economic, Political and Social Change in a Village of North Central India. Human Organization. 1952 Jun 1;11(2):5–12.
125. Sankhdher LM. Caste Interaction in a Village Tribe: An anthropological Case Study of the Tribes in Dhanaura Village in Mirzapur District of Uttar Pradesh. New Delhi: K. B. Publications; 1974.
126. McCauley AP. Balinese. In: Encyclopedia of World Cultures. Boston: Hall & Co; 1991.
127. Levinson D. Wogeo. In: Encyclopedia of World Cultures. Boston: G. K. Hall & Co; 1991;2:380–382.
128. Adams KJ. Work Opportunity and Household Organization among the Barama River Caribs of Guyana. Anthropos. 1979;1(2):219–222.
129. Forte J. The case of ‘the Barama river carbis of Guyana reestudied’. 1990;16.
130. Gillin JP. The Barama River Caribs of British Guiana. Vol. 14. The Museum; 1936.
131. Moore GW. Bhil Cultural Summary, New Haven. New Haven: Human Relations Area Files; 1965.
132. Nath YVS. Bhils of Ratanmal: An Analysis of the Social Structure of a Western Indian Community. Baroda: Maharajah Sayajirao University; 1960.
133. Naik TB. The Bhils: A Study. Delhi: Bharatiya Adimjati Sevak Sangh; 1956.
134. Whiteley W. Bemba and Related Peoples of Northern Rhodesia, Ethnographic Survey of Africa, East Central Africa. London: International African Institute; 1950.
135. Slaski J. Peoples of the Lower Luapula Valley. In: Bemba and Related Peoples of Northern Rhodesia. London: International African Institute; 1950:77–100.
136. Richards AI. The Political System of the Bemba Tribe of North-Eastern Rhodesia. In: African Political Systems. London: International African Institute; 1940:83–120.
137. Jenks AE. The Bontoc Igorot. Bulletin of the American Geographical Society. 1905;37(9):575.
138. Birket-Smith K. The rice cultivation and rice-harvest feast of the Bontoc Igorot. Munksgaard; 1952.
139. Jolly M. Soaring Hawks and Grounded Persons: The Politics of Rank and Gender in North Vanuatu. In: Big Men and Great Men: Personifications of Power in Melanesia. Cambridge: Cambridge University Press; 1991:48–80.
140. O’Leary TJ. Burusho Cultural Summary. New Haven, Conn: Human Relations Area Files; 1965.
141. Crocker WH. The Canela: An Ethnographic Introduction. Washington, D.C.: Smithsonian Institution Press; 1990. (Smithsonian Contributions to Anthropology; vol. 33).
142. Hicks GL, Speck FG. Cultural Persistence versus Local Adaptation: Frank G. Speck’s Catawba Indians. Ethnohistory. 1965;12(4):343.
143. Harrington MR. Catawba potters and their work. American Anthropologist. 1908 Jul 9;10(3):399–407.
144. Moore SF, Puritt P. The Chagga and Meru of Tanzania: East Central Africa Part XVIII. Vol. 18. Routledge; 1977.
145. Bergman R. Amazon Economics: The Simplicity of Shipibo Indian Wealth. New York: Dellplain Latin American Studies; 1980.
146. Sharpe B. Ethnography and a regional system: Mental maps and the myth of states and tribes in north-central Nigeria’. Critique of Anthropology. 1986;6(3):33–65.
147. Fitzgerald DG, Conley RJ. Cherokees. Portland: Graphic Arts Publishing; 2002.
148. Perdue T. The Cherokee. New York: Chelsea House; 1989.
149. Waldman C. Encyclopedia of Native American Tribes. New York: Checkmark; 1999.
150. Bogoras W. The Chukchee. Leiden: E. J. Brill; 1909.
151. Montag D, Kuch U, Rodriguez L, Müller R. The Lima Declaration on Biodiversity Climate Change: Contributions from Science to Policy for Sustainable Development. Convention of Biological Diversity. In: Secretariat of the Convention on Biological Diversity. Montreal; 2017. p. 1–156.
152. Clark AF, Phillips LC. Historical dictionary of Senegal. Metuchen: Scarecrow Press; 1994.
153. Abraao MB, Shepard GH, Nelson BW. Baniwa vegetation classification in the white-sand Campinarana habitat of the northwest Amazon, Brazil. Landscape ethnoecology Concepts of biotic and physical space. 2010;83–115.
154. Bureau of Native Affairs. Working papers in Dani ethnology, No.1, Bureau of Native Affairs hectograph. United Nations Temporary Executive Authority in West New Guinea-West Irian; 1962.
155. Heider KG. The Dugum Dani: A Papuan Culture in the Highlands of West New Guinea. New York: Viking Fund Publications in Anthropology; 1970.
156. Rappaport RA. Pigs for the ancestors. Ritual in the ecology of a New Guinea people. New Haven: Yale University Press; 1968.
157. Bremaud O, Pagot J. Grazing Lands, Nomadism, and Transhumance in the Sahel’. In: The Problems of the Arid Zone. Paris: UNESCO; 18,1962.
158. Briggs LC. Tribes of the Sahara. Cambridge: Harvard University Press; 1960.
159. Johnson DL. The Nature of Nomadism: A Comparative Study of Pastoral Migration in Southwestern Asia and North Africa. Chicago: University of Chicago; 1969.
160. Goddard I. Delaware. In: Handbook of North American Indians. Washington, D.C.: Smithsonian Institution; 1978:213–39.
161. Newcomb W. The Culture and Acculturation of the Delaware Indians’. Vol. 10. University of Michigan Museum of Anthropology: Ann Arbor; 1956.
162. Paulme D. Organisation sociale des dogon (Soudan Français). Paris: Éditions Domat-Montchrestien, F. Loviton & Cie; 1940.
163. Griaule M, Dieterlen G. The Dogon of the French Sudan. In: African Worlds: studies in the cosmological ideas and social values of African peoples. International African Institute: Oxford University Press; 1954: 83–110.
164. Marshall S, Das R, Pirooznia M, Elhaik E. Reconstructing Druze population history. Sci Rep. 2016 Dec;6(1):35837.
165. Herlihy PH. A cultural geography of the Embera and Wounan (Choco) Indians of Darien, Panama, with emphasis on recent village formation and economic diversification. Louisiana State University and Agricultural and Mechanical College; 1986.
166. Colin FL. Nosotros no solamente podemos vivir de cultura: Identity, Nature, and Power in the Comarca Emberá of Eastern Panama. [Ottawa, Canada]: Department of Geography and Environmental Studies Carleton University; 2010.
167. Brown P. Highland Peoples of New Guinea. Cambridge University Press; 1978.
168. Waddell E. How the Enga cope with frost: Responses to climatic perturbations in the Central Highlands of New Guinea. Hum Ecol. 1975 Oct;3(4):249–73.
169. Willis RG. The Fipa and related peoples of south-west Tanzania and North-east Zambia. In: Ethnographic Survey of Africa, East Central Africa. London: International African Institute; 1966. (15).
170. Sorenson ER, Gajdusek DC. Nutrition in the kuru region. 1. Gardening, food handling, and diet of the Fore people. Acta tropica. 1969;26:281–330.
171. Sorenson ER, Claessen HJM, du Torr BM, Griffith J, Hockings P, Jablonko A, et al. Socio-Ecological Change Among the Fore of New Guinea [and Comments and Replies]. Current Anthropology. 1972 Jun;13(3/4):349–83.
172. Hamilton-Reid L, Gajdusek DC. Nutrition in the Kuru region. Part II, a nutritional evaluation of traditional fore diet in Moke Village in 1957. Acta Tropica. 1969;26(4):332–3.
173. Huntingford GWB. The Galla of Ethiopia: The Kingdoms of Kafa and Janjero. London: International African Institute; 1955.
174. Earthy ED. 271. The Social Structure of a Gbande Town, Liberia. Man. 1936 Dec;36:203.
175. Schwab G. Tribes of the Liberian Hinterland. Cambridge: Peabody Museum; 1947.
176. Shillington K. Encyclopedia of African History 3-Volume Set. Routledge; 2013.
177. Olson JS. The Peoples of Africa: An Ethnohistorical Dictionary. Westport, Connecticut: Greenwood Press; 1996.
178. Ndanga DN. When the past becomes the future: aspects of cultural revitalization among the Gbaya in Bertoua, East-Cameroon. Universitetet i Tromsø; 2007.
179. Dozier EP. Hano: A Tewa Indian Community in Arizona. New York: Holt; 1966.
180. Joe S. The Pueblo Indians. San Francisco: Indian Historian Press; 1976
181. Rinehart W Ortiz A. Handbook of North American Indians. Vol. 9. Washington, D.C.: Smithsonian Institution; 1979.
182. Jacobs SE. Tewa Pueblos. In: Levinson D, Hockings P. Encyclopedia of World Cultures. Boston: G.K. Hall & Co; 1991.
183. Conklin HC. The relation of Hanunóo culture to the plant world. [New Haven (CT)]: Yale University; 1954.
184. Schwartz DW. Havasupai. In: Davis MB. The Gale Encyclopedia of Native American Tribes. Middle America, Detroit: Gale Research; 1998;113–116.
185. Bacon EE. The Inquiry into the History of the Hazara Mongols of Afghanistan. Southwestern Journal of Anthropology. 1951 Oct;7(3):230–247.
186. Ferdinand K. Preliminary Notes on Hazāra Culture:(The Danish Scientific Mission to Afghanistan. I Kommission hos Munksgaard; 1959, 37(5).
187. Poladi H. The Hazaras. Stockton: Moghal; 1989.
188. Matthews W. Ethnography and philology of the Hidatsa Indians. Vol. 7. US Government Printing Office; 1877.
189. West BA. Encyclopedia of the Peoples of Asia and Oceania. Infobase Publishing; 2010.
190. Kumar V. ‘Resettlement’-Adding New Languages in the Life of the Bhils and the Pawras of the West Central India’. International Journal of Innovations in TESOL and Applied Linguistics. 2019;4(2):1–11.
191. Lumholtz C. Unknown Mexico. Vol. 2. New York: Scribner’s; 1902.
192. Neurath J. Huicholes. México: CDI: PNUD; 2003.
193. Weigand PC. Differential Acculturation among the Huichol Indians. In: Themes of Indigenous Acculturation in Northwest Mexico. Tucson: University of Arizona Press; 1981:9–21.
194. Heidenreich CE. Huron. In: Handbook of North American Indians. New York: Smithsonian Institution; 1978:368–88.
195. Sagard G. The Long Journey to the Country of the Hurons. The Champlain Society; 1939.
196. Tooker E. An Ethnography of the Huron Indians 1615-1649. Midland, Ontario: Huronia Historical Development Council; 1967.
197. Trigger BG. The Huron Farmers of the North. New York: Holt, Rinehart and Winston; 1969.
198. Bateson G. Social Structure of the Iatmül People of the Sepik River (Concluded). Oceania. 1932;2(4):401–53.
199. Metraux R. Aristocracy and Meritocracy: Leadership among the Eastern Iatmul. Anthropological Quarterly. 1978 Jan;51(1):46.
200. Mead M. Public Opinion Mechanisms Among Primitive Peoples. Public Opinion Quarterly. 1937 Jul;1(3):5.
201. Sosis R. Ifaluk atoll: An ethnographic account. New Haven: Human Resources Area Files; 2005.
202. Burrows EG. From Value to Ethos on Ifaluk Atoll. Southwestern Journal of Anthropology. 1952 Apr;8(1):13–35.
203. Betzig L. Childcare on Ifaluk. Zeitschrift fur Ethnologie. 1989;18.
204. Bellwood PS, Fox JJ, Tryon DT, Australian National University, editors. The Austronesians: historical and comparative perspectives. Canberra: Dept. of Anthropology as part of the Comparative Austronesian Project, Research School of Pacific and Asian Studies, Australian National University; 1995:359
205. Barton RF. Ifugao Economics. University of California Publications in American Archaeology and Ethnology. 1922;15:385–446.
206. Conklin HC. Ethnographic Atlas of Ifugao: A Study of Environment, Culture, and Society in Northern Luzon. New Haven: Yale University Press; 1980.
207. Afigbo AE. Ropes of Sand: Studies in Igbo History and Culture. Ibadan and Oxford: Ibadan University Press and Oxford University Press; 1981.
208. Horton R. Stateless Societies in the History of West Africa. In: History of West Africa. London: Longman; 1976:72-113.
209. Isichei E. A History of the Igbo People. New York: St. Martin’s Press; 1976.
210. Onwuejeogwu MA. An Igbo Civilization: Nri Kingdom & Hegemony. London: Ethiope Publishing; 1981.
211. Uchendu VC. The Igbo of Southeast Nigeria. New York: Holt, Rinehart & Winston; 1965.
212. Jaspan MA. The Ila-Tonga Peoples of North-western Rhodesia. London: International African Institute; 1953.
213. Lewis MP, Simons GF, Fennig CD. Ethnologue: Languages of the World. Texas: SIL International; 2013.
214. Smith EW, Dale WM. The Ila-speaking Peoples of Northern Rhodesia. London: McMillan and Co. Ltd; 1920.
215. Wurm SA, Hattori S. Language Atlas of the Pacific Area. Vol. 2. Canberra: Australian Academy of the Humanities; 1983.
216. Börjeson L. Boserup backwards? Agricultural intensification as ‘its own driving force’in the Mbulu highlands, Tanzania’. Geografiska Annaler: Series B, Human Geography. 2007;89(3):249–67.
217. Börjeson L. A History under Siege: intensive agriculture in the Mbulu Highlands. Acta Universitatis Stockholminesis; 2004.
218. Snyder KA. The Iraqw of Tanzania. Negotiating rural development. New York: Westview Press; 2005.
219. Thornton RJ. Space, time and culture among the Iraqw of Tanzania. New York: Academic Press; 1980.
220. Damon FH. The Kula and Generalised Exchange: Considering Some Unconsidered Aspects of the Elementary Structures of Kinship. Man. 1980 Jun;15(2):267.
221. Damon FH. What Moves the Kula: Opening and Closing Gifts on Woodlark Island. In: The Kula: New Perspectives on Massim Exchange. Cambridge: Cambridge University Press; 1983:309–42.
222. LaRaw M. Towards a Basis for Understanding the Minorities of Burma: The Kachin Example. In: Southeast Asian Tribes, Minorities, and Nations. Princeton, N.J.: Princeton University Press; 1967:125–46.
223. Lehman FK. Introduction: Notes on Edmund Leach’s Analysis of Kachin Society and Its Further Applications. In: Social Dynamics in the Highlands of Southeast Asia: Reconsidering Political Systems of Highland Burma. Boston: Brill Acadmic Publishers; 2007.
224. Hanson O. The Kachins: Their Customs and Traditions. Rangoon: American Baptist Mission Press; 1913.
225. Friedman J. System, Structure, and Contradiction. Copenhagen: National Museum of Denmark; 1979.
226. Carrapiet WJS. The Kachin Tribes of Burma. Rangoon: Superintendent of Government Printing and Stationery; 1929.
227. Teklehaymanot T, Giday M. Ethnobotanical study of wild edible plants of Kara and Kwego semi-pastoralist people in Lower Omo River Valley, Debub Omo Zone, SNNPR, Ethiopia. J Ethnobiology Ethnomedicine. 2010 Dec;6(1):23.
228. Gurtong website [Internet]. Available from: www.gurtong.net
229. Henry J. Jungle People: A Kaingang Tribe of the Highlands of Brazil. New York: Vintage Books; 1941.
230. Hicks D. A Comparative Study of the Kaingang and Aweikoma of Southern Brazil’. University of Oxford; 1965.
231. Williams FE. Rain-Making on the River Morehead. The Journal of the Royal Anthropological Institute of Great Britain and Ireland. 1929 Jul;59:379.
232. Williams FE. Papuans of the Trans-Fly. Territory of Papua Anthropology Report no. 15. Oxford: Clarendon Press; 1936.
233. Ambler C. Kenyan Communities in the Age of Imperialism. New Haven: Yale University Press; 1988.
234. Cagnolo C. The Agikuyu: Their Customs, Traditions, and Folklore. Nyeri: The Mission Printing School; 1933.
235. Middleton J. The Central Tribes of the North-Eastern Bantu. London: International African Institute; 1953.
236. Muriuki GA History of the Kikuyu: 1500-1900. Nairoibi: Oxford University Press; 1974.
237. Serpenti L. Cultivators in the Swamps: Social Structure and Horticulture in a New Guinea Society (Frederik-Hendrik Island West New Guinea). Assen: Van Gorcum; 1965.
238. Stürzenhofecker G. Border crossings; Papua New Guinea models in Irian Jaya. Bijdr taal land volkenkd. 1991;147(2):298–325.
239. Gibbs JL. The Kpelle of Liberia. In: Peoples of Africa. New York: Holt; 1965:197–240.
240. Rinehart W. And Raymond G. Ethnologue: Languages of the World. 5th ed. Dallas: SIL International; 2005.
241. Holsoe SE, Lauer JJ. Who Are the Kran/Guere and the Gio/Yacouba? Ethnic Identifications along the Liberia-Ivory Coast Border. African Studies Review. 1976 Apr;19(1):139.
242. McEvoy FD. Understanding Ethnic Realities among the Grebo and Kru Peoples of West Africa. Africa. 1977 Jan;47(1):62–80.
243. Gragson TL. Fishing the Waters of Amazonia: Native Subsistence Economies in a Tropical Rain Forest. American Anthropologist. 1992 Jun;94(2):428–40.
244. Carneiro RL. Slash-and-burn cultivation among the Kuikuru and its implications for cultural development in the Amazon Basin. Caracas: Editorial Sucre; 1961.
245. Micheli I. Living a Kulango Life: Examples of Socialization under the Shadow of the Laasagyo’. International Journal of Agro-Asiatic Studies. 2016;20:257–66.
246. Micheli I. Son of the root: Djedwa Yao Kuman: Kulango healer and hunter. Trieste: EUT, Edizioni Università di Trieste; 2017:1-297.
247. Gordon RG. Ethnologue: Languages of the World. Dallas: SIL International; 2005.
248. Miracle M. Agriculture in the Congo Basin: Tradition and Change in African Rural Economies. Madison: University of Wisconsin Press; 1967.
249. Sato H. Hunting of the Boyela, Slash-and-Burn Agriculturalists, in the Central Zaire Forest. Kyoto University: African Study Monographs; 1983.
250. Vansina J. Finding Food and the History of Precolonial Equatorial Africa: A Plea. African Economic History. 1979;(7):9.
251. Lewis MP, Simons GF, Fennig CD. Ethnologue: Languages of the World. Dallas: SIL International; 2014.
252. Oliver DL. Studies in the Anthropology of Bougainville, Solomon Islands. Cambridge: Peabody Museum; 1949.
253. Sprenger G. From Power to Value: Ranked Titles in an Egalitarian Society, Laos. J of Asian Stud. 2010 May;69(2):403–25.
254. Sprenger G. Out of the ashes: Swidden cultivation in highland Laos. Anthropology Today. 2006 Aug;22(4):9–13.
255. Bunge FM, Cooke MW. Oceania: A Regional Study. Washington: U.S. Government Printing Office; 1984.
256. Hocart AM. Lau Islands, Fiji. Honolulu: Bernice P. Bishop Museum; 1929.
257. Thompson L. Southern Lau, Fij: An Ethnography. Honolulu: Bernice P. Bishop Museum; 1940.
258. Hanks LM, Hanks JR, Sharp L. Ethnographic Notes on Northern Thailand. 58th ed. Department of Asian Studies: Cornell University; 1965.
259. Douglas M. The Lele of the Kasai. International African Institute: Oxford University Press; 1963.
260. Douglas M. The Pattern of Residence among the Lele’. Zaïre. 1957;22(8):46–58.
261. Douglas M. The Lele of Kasai. In: African Worlds: studies in the cosmological ideas and social values of African peoples. International African Institute: Oxford University Press; 1954:1–26.
262. Morris J. Living with Lepchas: A Book about the Sikkim Himalayas. London: William Heinemann; 1938.
263. Siiger H, Rischel J. The Lepchas: Culture and Religion of a Himalayan People. Copenhagen: National Museum of Denmark; 1967. (Ethnographical Series).
264. Tobias M. Mountain People. Norman: University of Oklahoma Press. New York: Basic Books; 1967.
265. Powdermaker H. Life in Lesu: The Study of a Melanesian Society in New Ireland. New York: Norton; 1933.
266. Yin M. China’s Minority Nationalities. Beijing: Foreign Languages Press; 1989.
267. Hattingh PS. The Lobedu of the Transvaal’. South African Journal of African Affairs. 1975;5(2):73–5.
268. Mampeule PJ. A Short History of the Lobedu, Their Contact with Missionaries and the Origin and Development of the Christian Faith in Bolobedu 1600-1981. University of Limpopo; 2000.
269. Mönnig HO. The structure of Lobedu social and political organisation. African Studies. 1963;22(2):49–64.
270. Dietler M, Herbich I. Living on Luo time: Reckoning sequence, duration, history and biography in a rural African society. World Archaeology. 1993 Oct;25(2):248–60.
271. Evans-Pritchard EE. Luo Tribes and Clans. Rhodes-Livingstone Journal. 1949;7:24–40.
272. Ocholla-Ayayo ABC. The Luo Culture: A Reconstruction of the Material Culture Patterns of a Traditional African Society. Wiesbadem: Franz Steiner Verlag; 1980.
273. Spencer P. The Maasai of Matapato: A Study of Rituals of Rebellion. Manchester: Manchester University Press; 1988.
274. Gulliver PH. Social Control in an African Society: A Study of the Arusha, Agricultural Maasai of Northern Tanganyika. London: Routledge & Kegan Paul; 1962.
275. Hodgson DL. Pastoralism, patriarchy and history: changing gender relations among Maasai in Tanganyika, 1890–1940. J Afr Hist. 1999 Mar;40(1):41–65.
276. Spear T, Nurse D. Maasai Farmers: The Evolution of Arusha Agriculture. The International Journal of African Historical Studies. 1992;25(3):481.
277. Rigby P. Persistent Pastoralists. Nomadic Societies in Transition. Zed Books; 1985.
278. Spoehr A. Majuro, a Village in the Marshall Islands. Chicago: Field Museum of Natural History; 1949.
279. Tobin JA. Land Tenure in the Marshall Islands’. In: Land Tenure Patterns: Trust Territory of the Pacific Islands. Guam: Office of the High Commissioner; 1958.
280. Silverman MG. Disconcerting Issue: Meaning and Struggle in a Resettled Pacific Community. Chicago: University of Chicago Press; 1971.
281. Mambila society website [Internet]. [cited 2021 Mar 17]. Available from: http://www.mambila.info
282. Hurault J. Land crisis on the Mambila plateau of Nigeria, West Africa. Journal of biogeography. 1998;25(2):285–99.
283. Blench RM. Conflict and co-operation fulbe relations with the mambila and samba people of southern Adamawa. 1984;17.
284. Van Geluwe H. Mamvu-Mangutu et Balese-Mvuba. In: Ethnographic Survey of Africa, Central Africa Belgian Congo. London: International African Institute; 1957(3).
285. Fenn EA. Encounters at the Heart of the World: A History of the Mandan People. Hill and Wang; 2014.
286. Wood R. An interpretation of Mandan culture history. Bureau of American Ethnology Bulletin; 1967.
287. Cooper J. The Araucanians. In: Handbook of South American Indians The Andean Civilizations, Bureau of American Ethnology. Washington, D.C.: Smithsonian Institution. 1946;143(2):687–766.
288. Faron L. The Mapuche Indians of Chile. New York: Holt, Rinehart &Winston; 1968.
289. Dening G. The Marquesan Journal of Edward Roberts. Canberra: Australian National University Press; 1974.
290. Thomas N. Marquesan Societies: Inequality and Political Transformation in Eastern Polynesia. Oxford: Oxford University Press; 1990.
291. Hambly WD. The Ovimbundu of Angola. Anthropological Series. 1934;21(2):87–262.
292. Pospisil L. The Kapauku Papuans and Their Kinship Organization. 2022;19.
293. Pospisil L. Kapauku Papuan Economy. 67th ed. New Haven: Yale University Publications in Anthropology; 1963.
294. Pospisil L. The Kapauku Papuans of West New Guinea. New York: Holt, Rinehart and Winston; 1978.
295. Leach M. Rainforest Relations: Gender and Resource Use among the Mende of Gola, Sierra Leone. Washington, D.C.: Smithsonian Institution Press; 1994.
296. Little K. The Mende of Sierra Leone. London: Routledge & Kegan Paul; 1967.
297. Little K. The Mende in Sierra Leone’. In: African Worlds: studies in the cosmological ideas and social values of African peoples. Oxford University Press: International African Institute. 1954:111–37.
298. Ziran B. A Happy People: The Miaos. Beijing: Foreign Languages Press; 1988.
299. Schein L. The Dynamics of Cultural Revival among the Miao in Guizhou. In: Ethnicity and Ethnic Groups in China. Hong Kong: Chinese University Press. 1989:199–210.
300. Turner WY. The Ethnology of the Motu. The Journal of the Anthropological Institute of Great Britain and Ireland. 1878;7:470–99.
301. Dutton TE. The Hiri in History: Further aspects of long distance Motu trade in Central Papua, Pacific. Australian National University: cific Research Monograph. 1982;(8).
302. Groves M. Western Motu Descent Groups. Ethnology. 1963 Jan;2(1):15.
303. Boonzaier E, Malherbe C, Smith AB, Berens P. The Cape Herders: A History of the Khoikhoi of Southern Africa. New Africa Books; 1997.
304. Carstens WP. The Social Structure of a Cape Coloured Reserve. Cape Town: Oxford University Press; 1966.
305. Kluckhohn C, Leighton D. The Navaho. Cambridge: Harvard University Press; 1946.
306. Locke RF. The Book of the Navajo. Los Angeles: Mankind Publishing Co; 1976.
307. Ortiz A. Handbook of North American Indians. Vol. 10. Washington, D.C.: Smithsonian Institution; 1983.
308. Underhill R. The Navajos. Norman: University of Oklahoma Press; 1956.
309. Reddy S. The Nicobar Islands, Cultural Choices in the aftermath of the Tsunami Simron Jit Singh’. Indian Anthropologist. 2006;36(2):183–6.
310. Lehman O, Verlag C, Wildenberg M, Singh SJ. Integrated Modelling and Scenario Building for the Nicobar Islands in the Aftermath of the Tsunami’. In: Human-Nature Interaction in the Anthropocene: Potentials of Social-Ecological Systems Analysis. New York: Routledge; 2012:161–89.
311. Mckenny MG. The Social Structure of the Nyakyusa: a Re-evaluation. Africa. 1973 Apr;43(2):91–107.
312. Reddy S. The Nicobar Islands, Cultural Choices in the aftermath of the Tsunami Simron Jit Singh’. Indian Anthropologist. 2006;36(2):183–6.
313. Lehman O, Verlag C, Wildenberg M, Singh SJ. Integrated Modelling and Scenario Building for the Nicobar Islands in the Aftermath of the Tsunami’. In: Human-Nature Interaction in the Anthropocene: Potentials of Social-Ecological Systems Analysis. New York: Routledge. 2012:161–89.
314. Lewis MP. Ethnologue: Languages of the World. Dallas: SIL International; 2009.
315. Langlands BW. The Population Geography of Bunyoro District. 35th ed. Uganda: Department of Geography Mackerere University; 1971.
316. Oliver R. The Traditional Histories of Buganda, Bunyoro, and Nkole. The Journal of the Royal Anthropological Institute of Great Britain and Ireland. 1955;85(1/2):111.
317. Beattie J. The Nyoro State. Oxford: Clarendon Press; 1971.
318. Dorsey JO. Omaha Sociology. Bureau of American Ethnology. 1884;3:205–307.
319. Fortune RF. Omaha Secret Societies. New York: AMS Press; 1969.
320. Mead M. The Changing Culture of an Indian Tribe. New York: Columbia University Press; 1932.
321. O’Shea J. and Ludwickson, J. Archaeology and Ethnohistory of the Omaha: The Big Village Site. Lincoln: University of Nebraska Press; 1992.
322. New Guinea Research Unit. Orokaiva Papers. New Guinea Research Bulletin 13. Canberra: Australian National University Press; 1966.
323. Schwimmer E. Reciprocity and Structure: A Semiotic Analysis of Some Orokaiva Exchange Data. Man. 1979 Jun;14(2):271.
324. Williams FE. Orokaiva Society. London: Oxford University Press; 1930.
325. Chiang BD. Paiwan Qelu. Expedition. 1986;28(3):46–54.
326. Chiang BD. House and social hierarchy of the Paiwan. University of Pennsylvania; 1993.
327. Hanbury-Tenison RA Question of Survival for the Indians of Brazil. London: Angus & Robertson; 1973.
328. Métraux A. The Paressi. In: Handbook of South American Indians. The Tropical Forest Tribes. 1958:349–60.
329. Moi FP, Morales WF. Archaeology and Paresi Cultural Heritage. Indigenous Peoples and Archaeology in Latin America. 2011;4:315–32.
330. Hyde GE. Pawnee Indians. Denver: University of Denver Press; 1951.
331. Murie JR. Pawnee Indian Societies. Anthropological Papers of the American Museum of Natural History. 1914;11(4).
332. Svingen OJ. The Pawnee of Nebraska: Twice Removed. American Indian Culture and Research Journal. 1992;16(2):121–37.
333. Borofsky R. Making history: the creation of traditional knowledge on Pukapuka, a Polynesian atoll. University of Hawaii; 1982.
334. Needham R. A Structural Analysis of Purum Society. American Anthropologist. 1958 Feb;60(1):75–101.
335. Gogoi NK. Issues in the purum debate. [Department of Anthropology]: North-Eastern Hill University; 1989.
336. Cauquelin J. Aborigines of Taiwan: the Puyuma: from headhunting to the modern world. Routledge; 2004.
337. Chen WT. The making of a’community’: An anthropological study among the Puyuma of Taiwan. SOAS University of London; 1998.
338. Bash KW, Bash-Liechti J. The Qashqai. Berlin: Springer; 1987.
339. Gharakhlou M. A study of cultural changes among the Qashqai tribes in Iran’. Researches in Geography. 2006;56:1–11.
340. Bee R. Crosscurrents along the Colorado: The impact of government policy on the Quechan indians. University of Arizona Press; 2020.
341. Myers KP, Brunstrom JM, Rogers PJ, Holtzman JD. Portion size influences intake in Samburu Kenyan people not exposed to the Western obesogenic environment. Appetite. 2019 Feb;133:212–6.
342. Holtzman J. Samburu. The Rosen Publishing Group; 1995.
343. Teixeira P, Brasil M, Silva EM da. Demografia de um povo indígena da Amazônia brasileira: os sateré-mawé. Rev bras estud popul. 2011 Dec;28(2):429–48.
344. Benjamin G. Austroasiatic Subgroupings and Prehistory in the Malay Peninsula. 2022;93.
345. Fleuret PC. Farm and market: a study of society and agriculture in Tanzania. University of California; 1984.
346. Von Haimendorf C. Sherpas of Nepal: Buddhist Highlanders. Berkeley: University of California Press; 1964.
347. Arens W. The Divine Kingship of the Shilluk: A Contemporary Reevaluation’. Ethnos. 1979;44:167–81.
348. Evans-Pritchard EE. The Divine Kingship of the Shilluk. Cambridge: Cambridge University Press; 1948.
349. Howell PP. The Shilluk settlement. Sudan Notes and Records. 1941; 24:47-67.
350. Lienhardt RG. The Shilluk of the Upper Nile. In: African Worlds. London: Oxford University Press. 1954:138–63.
351. Beach DN. The Shona and Zimbabwe, 900-1850. Gweru: Mambo Press; 1980.
352. Bourdillon MFC. The Shona Peoples. Gweru: Mambo Press; 1987.
353. Ellert H. The Material Culture of Zimbabwe. Harare: Longman; 1984.
354. Gelfand M. Growing Up in Shona Society. Gweru: Mambo Press; 1979.
355. Salisbury RF. The Siane of the Eastern Highlands’. In: Gods, Ghosts, and Men in Melanesia. New York: Oxford University Press; 1965:50–77.
356. Salisbury-Rowswell RF. Economic change among the Siane tribes of New Guinea. 1957.
357. Salisbury RF. The Siane Language of the Eastern Highlands of New Guinea. 1956;35.
358. Harding TG. A history of cargoism in Sio, north-east New Guina. Oceania. 1967 Sep;38(1):1–23.
359. Groves WC. The natives of Sio island, south-eastern New Guinea: a study in culture-contact. Oceania. 1934 Sep;5(1):43–63.
360. Gray RF. The Sonjo of Tanganyika: An Anthropological Study of an Irrigation-Based Society. International African Studies: Oxford University Press; 1963.
361. Kopytoff I. The Suku of Southwestern Congo. In: The Peoples of Africa. New York: Holt, Rinehart & Winston. 1988:441–78.
362. Hilmiati N, Budiwiranto B, van de Fliert E. Gender, ethnicity and engagement: Uptake strategies for smallholder cattle farming innovation in West Nusa Tenggara, Indonesia. 2017;9.
363. Hidayah Z. A Guide to Tribes in Indonesia: Anthropological Insights from the Archipelago. Springer Nature; 2020.
364. Wagley C. Welcome of Tears: The Tapirapé Indians of Central Brazil. New York: Oxford University Press; 1977.
365. Cline W. The Teda of Tibesti, Borkou, and Kawar in the Eastern Sahara’. 1950;(12).
366. Kramer KSC. The Effects of the Drought on the Teda of the Libyan Tibesti. University of Benghazi; 1975.
367. Nimuendajú C. The Tukuna. Berkeley and Los Angeles: University of California Press; 1952.
368. Firth R. We, The Tikopia: A Sociological Study of Kinship in Primitive Polynesia. London: Allen & Unwin; 1936.
369. Kirch PV, Yen DE. Tikopia: The Prehistory and Ecology of a Polynesian Outlier. Honolulu: Bishop Museum Press; 1982.
370. Bohannan L, Bohannan P. The Tiv of Central Nigeria. London: International African Institute; 1953.
371. Bohannan P, Bohannan L. Tiv Economy. Evanston, Ill. Northwestern University Press; 1968.
372. Rivers WHR. The Todas. London: Macmillan; 1906.
373. Walker AR. Toda. In: Encyclopedia of World Cultures. Boston: G. K. Hall & Co. 1992; (3).
374. Walker AR. The Toda of South India: A New Look. Delhi: Hindustan Publishing Corporation; 1986.
375. Weiner AB. The Trobrianders of Papua New Guinea. New York: Holt, Rinehart and Winston; 1988.
376. Bernus E. Touaregs nigeriens: Unité culturelle d’un peuple pasteur. Paris: Éditions de l’Office de la Recherche Scientifique et Technique d’Outre-Mer; 1981.
377. Lhote H. Touareg du Hoggar. Paris: Payot; 1953.
378. Nicolaisen J. Ecology and Culture of the Pastoral Tuareg. Copenhagen: National Museum of Copenhagen; 1963.
379. Palmer HR. The Tuareg of the Sahara II. African Affairs. 1932 Jul;31(124):293–308.
380. Baier S, Lovejoy PE. The Tuareg of the Central Sudan: graduations in servility and the desert age (Niger and Nigeria). In: Slavery in Africa; historical and anthropological perspectives. Leiden University Press; 1977. p. 391–411.
381. Rodd L. The People of the Veil. London: Anthropological Publications; 1926.
382. Rasmussen SJ. Tuareg. 1995,(9).
383. Métraux A. The Tupinamba. US Government Printing Office; 1987.
384. Carrier J, Carrier A. Wage, Trade, and Exchange in Melanesia: A Manus Society in the Modern Stat. Berkeley: University of California Press; 1989.
385. Levinson D. Barama River Carib’. In: Encyclopedia of World Cultures: South America. Boston: Hall & Co; 1991.
386. Jochelson W. The Yakut. 33rd ed. New York: American Museum of Natural History Anthropological Papers; 1933.
387. Tokarev SA, Gurvich IS. The Yakuts. In: Peoples of Siberia. Chicago: University of Chicago Press; 1964: 547–70.
